# A Comprehensive Survey on Emerging Assistive Technologies for Visually Impaired Persons: Lighting the Path with Visible Light Communications and Artificial Intelligence Innovations

**DOI:** 10.3390/s24154834

**Published:** 2024-07-25

**Authors:** Alexandru Lavric, Cătălin Beguni, Eduard Zadobrischi, Alin-Mihai Căilean, Sebastian-Andrei Avătămăniței

**Affiliations:** 1 Department of Computers, Electronics and Automation, Stefan cel Mare University of Suceava, 720229 Suceava, Romania; lavric@usm.ro (A.L.); catalin.beguni@usm.ro (C.B.); eduard.zadobrischi@usm.ro (E.Z.); 2 Integrated Center for Research, Development and Innovation in Advanced Materials, Nanotechnologies and Distributed Systems for Fabrication and Control, Stefan cel Mare University of Suceava, 720229 Suceava, Romania; sebastian.avatamanitei@usm.ro; 3 East European Border Scientific and Technological Park, 725500 Siret, Romania

**Keywords:** AI-assisted VLC, blind person assistance, visually impaired assistance solutions, visually impaired person, visible light communications, wearable devices, wearable sensors

## Abstract

In the context in which severe visual impairment significantly affects human life, this article emphasizes the potential of Artificial Intelligence (AI) and Visible Light Communications (VLC) in developing future assistive technologies. Toward this path, the article summarizes the features of some commercial assistance solutions, and debates the characteristics of VLC and AI, emphasizing their compatibility with blind individuals’ needs. Additionally, this work highlights the AI potential in the efficient early detection of eye diseases. This article also reviews the existing work oriented toward VLC integration in blind persons’ assistive applications, showing the existing progress and emphasizing the high potential associated with VLC use. In the end, this work provides a roadmap toward the development of an integrated AI-based VLC assistance solution for visually impaired people, pointing out the high potential and some of the steps to follow. As far as we know, this is the first comprehensive work which focuses on the integration of AI and VLC technologies in visually impaired persons’ assistance domain.

## 1. Introduction

Blindness is a disorder characterized by the partial or complete loss of vision. Generally, blindness is caused by different causes, such as genetic factors, injuries to the eyes or brain, illnesses such as glaucoma or cataracts, infections, age-related conditions, or neurological disorders [1,2]. In its worst cases, blindness is associated with no light perception. In such conditions, blind people perceive the environment through their other senses [3,4,5] or by having support from technologies in order to improve their quality of life. Blindness considerably affects a person’s ability to execute daily actions and relate with their surroundings. From this perspective, performing regular activities such as reading and writing, traveling in various new areas, and accessing information become very challenging. Moreover, as a consequence of the previously mentioned facts, blindness can affect social interactions of all kinds [6], leading in turn to low self-esteem, isolation, depression and psychological distress [7], poor quality of life, and unhealthy habits [8]—and, in life or death situations, such as in emergency response when a fire starts, their actions are significantly affected [9]. Eye problems also affect persons’ employment rate and their income, putting a social burden on them and on their families [10].

Worldwide, about 0.5% of the population is affected by severe visual impairment and blindness [11,12]. Moreover, despite the significant progress achieved in ophthalmology, it is expected that the number of persons affected by such issues will significantly increase in the next decades. These negative perspectives are motivated by population growth and aging [12]. As eye problems affect human lives in a significant manner, appropriate solutions must be designed and investigated by the research community. On one hand, these solutions should be focused on prevention, early eye disease detection and treatment, and on the other hand, the efforts should be oriented to quality-of-life improvement for those who are already affected by severe visual impairment and blindness.

From the earliest times, people have been concerned about blind people and have tried to help them in various ways, laying the early foundations for assistive solutions and later for assistive technologies. Starting with white canes, guide dogs, the Braille alphabet, and mobile apps that provide access to digital information in a recognizable format, we are on our way to the point where Artificial Intelligence (AI) and Machine Learning (ML) tools will be used as personal assistants, able to read text, identify objects, environments, and people, understand situations, and provide complex real-time assistance [13,14]. From this perspective, in a world increasingly driven by technology and innovation, it is imperative to ensure that technological progress meets the needs and well-being of all members of society, including people with severe visual impairment. 

In this context, the main purpose of this article is to shed light on the current solutions aimed to assist blind individuals, point out their efficiencies, and debate potentially new technologies and methods that can further improve their self-reliance and quality of life. Different from other works or existing survey articles which address blind persons’ issues and which summarize existing technologies and solutions [15,16,17,18], this article aims to put the spotlight on the potential of two new technologies and to evaluate and debate their potential in improving blind persons’ quality of life. Thus, as AI and ML technologies are rapidly developing, they hold the potential to provide significant benefits, starting from early eye disease detection, and going to the point where AI is used to understand the blind persons’ needs, perceive the environment, and translate it in a suitable manner, in accordance with the persons’ expectations. 

AI algorithms are paving the way to new support systems for visually impaired people. AI techniques for blind persons integrate a wide range of technologies and applications designed to assist those with visual impairments in navigating their environment, accessing information, and performing everyday tasks more independently. In addition, this article aims to discuss the use of Visible Light Communications (VLC) technology in assisting the blind due to its unique characteristics, highlighting its potential to enhance mobility and facilitate access to wireless communications and beyond. By relying on the visible light spectrum and a widely available lighting network, VLC can support high data rate information exchange and connectivity and accurate indoor positioning, and offer navigational support. 

Moreover, as VLC is considered to be highly compatible with the Integrated Communications and Sensing (ICS) concept [19,20,21], additional features can and will be added, providing improved capabilities in environmental sensing. Consequently, VLC can enable blind people to travel more safely and confidently in complex environments. Therefore, unlike any existing survey article, this work aims to highlight the potential of VLC to support innovative, trustworthy, and cost-effective assistance solutions aimed at improving the independence and quality of life of blind people. Finally, this work aims to discuss how AI and VLC can be integrated into a comprehensive assistive solution that can provide superior performance.

In the context presented above, this article comes with a three-folded contribution. Firstly, this work provides a comprehensive analysis focused on commercially available solutions aimed at assisting blind and severe visually impaired persons in their daily activities. This section briefly describes their purpose, applicability, and characteristics, emphasizing their main advantages and pointing out their use cases and scenarios in which they could be most helpful.

Secondly, this article provides a comprehensive analysis related to the early identification of eye problems based on AI and ML techniques. This section summarizes the key AI algorithms that can be used to detect eye disease, enabling early treatment and sight preservation.

Thirdly, after presenting the characteristics of existing assistance solutions, this work moves forward by debating the benefits that could be brought to light by the use of VLC technology in blind persons’ assistance, pointing out that the characteristics of VLC could address many of the issues. Therefore, the article provides a review emphasizing that VLC is at the point of making the transition from a potentially useful technology to a technology which is ready to be deployed, and provides a pathway related to the integration of AI and VLC technologies as a superior solution in blind people’s assistance. Combined, these tools provide advanced, cost-effective solutions that enable blind individuals to travel unfamiliar environments safely and efficiently. As far as we know, this work is one of the most comprehensive studies that lights the path toward VLC integration with AI innovations in blind persons’ assistance applications.

In conclusion, the main contribution of this work is to provide unique perspectives on systems and applications for visually impaired persons’ assistance, considering the following three technology pillars:

(i) VLC technology enhancement for personal assistance;
(ii) AI enhancement for VLC data communication optimization;
(iii) AI for diagnostic systems that ultimately preserve vision.

The main purpose of this work is to emphasize the potential of, and to stimulate research activities in, the area of visually impaired person assistance systems.

The remainder of this article is structured as follows. Section 2 provides a summary of some of the most relevant commercial systems aimed for visually impaired persons’ assistance. Following, Section 3 provides a brief description of VLC and AI technologies, aiming to provide some of the reasons for which these technologies could be used in visual impaired persons’ assistance. Considering the importance of early detection, Section 4 focuses on the use of AI in early eye disease detection. Then, Section 5 presents some VLC systems designed for blind persons’ assistance. Based on the arguments provided in Section 3, Section 4 and Section 5, Section 6 aims to present a discussion focused on the future research perspectives related to this topic. Finally, Section 7 provides the conclusions of this work and final discussions.

## 2. Systems and Solutions to Assist Visually Impaired Persons 

**Figure 1:**
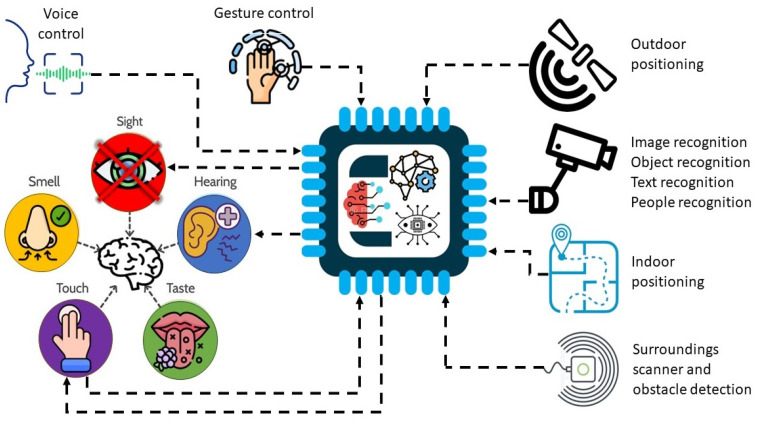
Illustration of the principles supporting visually impaired assistance systems: information that should have been received by sight is obtained based on different ambient perception sensors, processed and analyzed, and then the relevant content is provided to the user through one of the other senses (i.e., mainly hearing and touch).

Considering the major impact blindness has on daily life, human society has struggled to develop solutions that can improve visually impaired persons’ daily experience. Therefore, different technologies have been developed with the purpose of providing them with information that cannot be preserved by sight. The basic idea behind this principle is to replace the information provided by sight with information received and analyzed by different technical solutions which is then converted into a stimulus that can be received by a Visually Impaired Person (VIP). Figure 1 aims to provide an illustration of this concept, suggesting the idea that although it cannot replace sight, technology can be there to compensate part of the viewing experience.

The assistive devices can be placed in four categories [22]: (i) detection devices, (ii) navigation devices, (iii) devices for daily activities, and (iv) hybrid devices with multiple functions, which are increasingly seen nowadays. This subsection aims to present a brief review of some of the most important systems dedicated to assist visually impaired people. These devices use various sensors to collect information about the environment and provide feedback to VIPs through sound [23], haptic means, or both. As shown in [23], VIPs are able to recognize various locations, objects, or setups with the help of specific sounds, instead of using voice guidance. A summary of some of the latest devices and apps is presented in Table 1.

**Figure 2:**
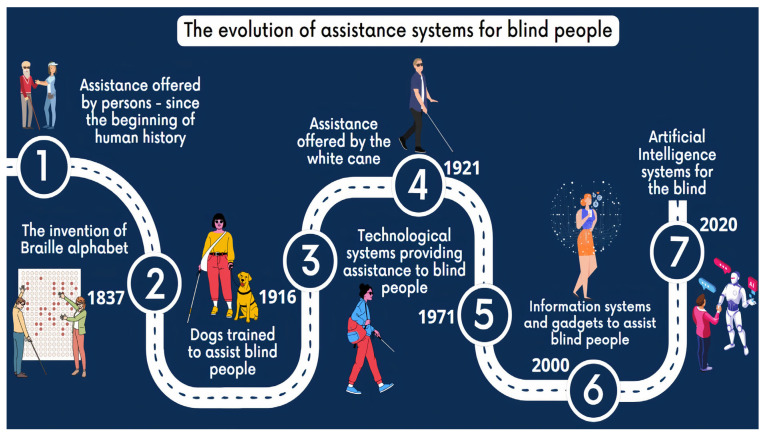
Illustration showing the evolution of visual impairment solutions.

One basic need for every human being is to be able to go to different places, and one difficulty for a blind person is to detect obstacles in his path. Nevertheless, for a comprehensive image (Figure 2), it should be remembered that the assistance of visually impaired persons started with the traditional human guide. This was the first visually impaired assistance method since the beginning of human history. Although it can be considered as highly reliable, not every time is a person able to assist the VIP, and not every VIP can afford professional help. Moreover, the human guide has the disadvantage of not offering the user the sense of freedom, making him feel like an assisted person. Another traditional method is based on guide dogs, trained to guide people with disabilities. Although quite efficient in personal assistance and in providing a mental comfort, this dog training requires quite some time, while having the disadvantage that the dogs require attention (i.e., feeding, walking, and cleaning) that can be difficult for some visually impaired people. A white cane is extensively used by VIPs as a detection tool due to its affordability, but has its drawbacks on sand or snow. In order to provide access to information in a VIPs day-to-day activity, an alphabet writing system was perfected in 1837 by Louis Braille, which was based on a tactile code.

Starting with the Digital Revolution, new technologies and inventions paved the way for the appearance of improved devices that ease the lives of visually challenged people. These technologies were first integrated in standalone devices. Therefore, the white cane was complemented in 1990 by the invention of the Hoople by Clive Ellis and Tony Larkin, which was designed to work on any terrain.

Once the Internet Age took the world by storm, new devices capable of assisting blind people appeared, including connectivity options and the integration of new artificial intelligence technologies. Since smartphones are now almost indispensable in everyone’s life, more and more assistive applications have started to be developed, transforming this device into an essential tool for most VIPs.

Navigation systems for blind and visually impaired individuals are referred to by various terms in the scientific community, including assistive navigation systems, visual substitution navigation systems, traveling aid systems, and walking assistants [24]. Regardless of the terminology, the ultimate goal is to assist blind individuals in three essential areas: (i) positioning, meaning the person’s position in relation to surrounding objects; (ii) localization, which is the person’s location on a map; (iii) and navigation, which provides directions to get from one point to another. The equipment that can help such individuals is quite diverse but generally relies on a few basic features: input sensors to collect data about the environment, beacons for orientation and localization, data processing systems, and user interfaces [25]. To this end, many manufacturers have focused on developing applications for smartphones, as these devices already have the necessary tools for monitoring the surrounding environment, such as microphones, cameras, touch screens, accelerometers, gyroscopes, magnetometers, and Global Positioning System (GPS) hardware, offering various methods of user interaction, including audio signals and haptic feedback (vibrations), but also computational capability and connectivity with other devices through WiFi, Bluetooth, or Near Field Communication (NFC). Several examples of assistive devices and applications developed for smartphones can be found in Table 1.

In order to sense the world around, VIPs rely basically on two senses: tactile and audio. Based on these capabilities, vibration guidance systems use various sensors for reading the environment and vibrators for feedback, in order to guide people with disabilities. An example of such a system is Wayband [26], which guides users to their destinations using only vibrations from a smart band connected with a mobile app (HapticNav). Sound guidance systems have become a viable alternative, especially since most people with disabilities use a variety of gadgets. BlindSquare [27] is an example of such an application that uses the smartphone as a user interface. It emits audible instructions, reading the surroundings’ text information, and also has Global Positioning System (GPS) hardware for navigation purposes in outdoor conditions. In order to help a VIP inside buildings, BlindSquare has the ability to detect Bluetooth beacons for Beaconing Position System (BPS) and read QR codes for orientation purposes.

In order to capture information from the environment, a plethora of sensors are used nowadays to collect data in an assistive device. Laser detection technology, such as LiDAR and laser rangefinders, can be used in guidance equipment, improving the precision of distance measurements for these devices [28]. This technology uses a laser as a signal source, emitting a laser beam that strikes obstacles and then is reflected back and received by a photodetector, allowing the distance between the laser sensor and the obstacle to be measured. The shape of obstacles can be accurately determined based on the amplitude, frequency, and phase of the reflected signal. Apart from all these advantages, there are also challenges, such as dealing with strong optical noise or transparent obstacles, so this type of detection is still in the development phase. Infrared guidance systems have drawn a lot of interest but, at the moment, ultrasonic technology is preferred, because it offers better range detection [29]. Therefore, in order to compensate for each other’s drawbacks, a combination of LiDAR and ultrasonic technology is used by Ara from Strap Tech to continuously scan the surroundings [30]. Each sensor offers distinct advantages, providing a comprehensive view of the environment, identifying obstacles based on their height level: top, mid, and low.

At the same time, many devices designed for assisting VIPs can be connected to a smartphone in order to be controlled, or even vice versa, to have control over the phone, as is the case with some smart canes that integrate keys and touchpads. One such example is WeWalk Smart Cane [31]. The device can be connected to a smartphone, providing precise location identification and step-by-step navigation instructions. Audible cues can be provided either directly from the stick or through the app. Customizable options allow directions to be given either with “right and left” instructions or from a clockwise approach. The WeWalk Smart Cane is compatible with iOS and Android, using its associated app. For users with low vision, the app provides various facilities, including color inversion and text size variation. In order to control the connected smartphone, the WeWalk cane incorporates a touchpad, allowing the user to keep the phone in the pocket and freeing his other hand. 

Other types of assistive devices are designed to help blind people in their daily tasks. In this regard, there are software applications capable of converting text to audio, and vice versa. Over the past few decades, synthesized speech has steadily evolved and improved, becoming nearly omnipresent in various applications, from virtual assistants to smartphone and tablet reading aids, especially for visually impaired individuals through text-to-speech conversion. A software application widely used by blind people is NVDA [32] and can be used for free on Windows operating systems. This program, available in 15 international languages, offers assistance to blind people by reading the computer screen. Another highly regarded application is Text to Speech TTS Voice Dream [33] (former Voice Dream Reader), ideal for opening and reading aloud a wide range of file formats, including PDFs, Microsoft Word files, PowerPoint presentations, web articles, and various e-book formats. Users can select from a variety of voices, including premium options, in over 20 languages. The app provides extensive navigation options, allowing users to navigate by sentence, paragraph, page, and chapter. The application has many other options but is only available for iOS and macOS.

Seeing AI [34] is a mobile application developed by Microsoft, designed to help visually impaired or partially sighted people improve their daily lives through technology use. The application can be used on iOS or Android mobile devices and is free to download and use. The main features of the Seeing AI application include the ability to recognize and read text from various sources, as well as to recognize objects and people. The information is transmitted to the user using a voice synthesizer. This application can read text from books, magazines, or other printed documents, as well as text on screens, but can also read QR codes. It can recognize text from images, including text from pictures on social media or messaging apps. The ability to recognize objects and people can be useful in many situations. For example, users can use the app to recognize and identify products in supermarkets or to identify people in social or professional environments. Audio descriptions for images recognized by the app can also be useful in many situations. For example, the app can describe a landscape or an object in a photo, giving users a clearer idea of what’s in the image. The Seeing AI application can also be customized according to user preferences. They can choose from multiple voices and set the app’s speaking speed. They can also set the app to provide haptic feedback via vibrations, allowing users to identify when the device is in a certain mode or when an object has been recognized.

A subscription-based service through the Aira application [35] provides assistance with navigation and object recognition by calling a live agent. Aira assistants can provide indications and communicate with the user via an audio/video connection through the smartphone. The user can transmit real-time images through the on-line connection and the agent can offer a wide range of services, including assistance in social and professional environments, such as office buildings, airports, and other public places. Users may use the Aira Service to request assistance in reading text, including text from books, screens, or other sources. The request for assistance can be made at any time of the day, regardless of location. Users can request assistance navigating unfamiliar buildings or finding specific locations within them. The Aira platform is currently available in the United States and a few other countries, and users can subscribe to monthly or yearly services, but the costs can be too high for other areas of the world.

GoodMaps Explore [36] can locate the user’s position inside a building with an accuracy down to a meter, by analyzing geo-referenced images through a camera-based positioning system. This is a substantial improvement over the accuracy provided by GPS (8–10 m outdoors), Bluetooth trilateration (4–5 m), or other techniques. Another advantage of this technique is that building owners can update and customize their indoor maps, ensuring that POI data is always up to date. Such an approach also allows the owner of the place to offer virtual tours inside the building. Another interesting feature is that the user can call Be My Eyes [37] from this application, which connects a blind user with a sighted volunteer. This is another application, free of charge, that can run independently on iOS and Android devices. Using the smartphone’s camera and microphone, the registered volunteer can provide access to additional data and information when visual assistance is needed.

WayAround [38] is an app based on the NFC read/write that helps visually impaired users identify and organize objects through smart tags. Tags can be programmed via a mobile app and read via a mobile reader. WayAround pricing starts from USD 74.99 for starter packs.

Concerning the standalone systems dedicated to assisting people with visual impairments, there are several types of devices and technologies that can support some of the vision problems, and improve these persons’ access to information, with the purpose of providing a decent day-to-day experience. Many of these devices are based on readers, touch screens, electronic magnifiers, audio headphones, Braille devices, Wi-Fi communications, mime and gesture control, and many others. Thus, concepts capable of reading the text on a screen and later converting it into an audio signal or Braille alphabet are becoming more and more common as part of many solutions. Based on such devices, visually impaired people can surf the Internet, read documents or emails, and also use other software applications, such as social networks. If we refer to people with partial sight problems, the electronic magnifying glass is one handy and widely used solution that enables them to adjust the font size in the text, making it much more legible. On the other hand, the most common solution used by people with visual impairments is based on audio headphones which are used to provide verbal feedback and guidance in unfamiliar or crowded areas.

The use of Braille devices adjusts the process of reading texts based on their conversion into a tactile writing system. These devices can also be connected to other gadgets or computers in order to play documents, books, and other types of content. The number of companies that produce such devices dedicated to people with disabilities grows from one year to another, but the most important ones remain: Freedom Scientific (producer of screen readers, electronic magnifiers, and also other types of devices), HumanWare (producer focused on Braille devices, but also technologies dedicated to the area of visually impaired people), BlindShell (producer of a special smartphone concept for visually impaired people), and OrCam (producer of a portable device capable of reading text content and object recognition). 

OrCam MyEye 3 Pro [39] is a portable system intended for visually impaired or partially sighted people, capable of helping the VIP to move in dynamic or unfamiliar places (buildings, offices, or crowded spaces). The system uses AI algorithms for the recognition and identification of objects and characters, providing the user with the information with the help of a speaker or an audio receiver. The OrCam MyEye device is small and easy to carry, being designed to clip onto the user’s eyeglass frames. It is equipped with a camera and processor, and one of the innovative functions is the hand gesture control function. One of the notable features of OrCam MyEye is its ability to read text in real time. This includes not only text written on paper, but also text on screens, as well as text from various media, such as restaurant menus, traffic signs, or product labels in stores. OrCam MyEye can also recognize objects and people, including faces, and provide the user with additional information about them. OrCam MyEye can also provide real-time navigation instructions. The OrCam MyEye device is controlled via a mobile app, which allows users to customize settings and receive software updates to improve device performance. OrCam MyEye can also be useful for people with cognitive or learning disabilities. They can benefit from the device’s ability to read text aloud, enabling them to understand and process information more efficiently.

Brailliant [40] is part of a range of standalone devices, developed by HumanWare, to help visually impaired people access information. These portable and easy-to-use devices allow users to read, write, and communicate through the Braille system. Brailliant is available in several sizes, from portable and easy-to-use devices to larger devices with multiple Braille lines, making them ideal for a wide range of users with different needs. Brailliant devices come with multiple features and options for customization, allowing users to tailor their experience to their preferences. These features include options to adjust vibration level and response speed. The devices are also equipped with a wide range of customizable buttons and functions to provide an intuitive and easy user experience. The devices are built with the latest technology and are also compatible with a variety of devices such as mobile phones, tablets, and computers. A wide range of connectivity options, including Bluetooth and Wi-Fi, allow users to connect to devices such as mobile phones and computers.

Victor Reader Stream 3, another device from HumanWare [41], is a portable digital audio player, specially designed for people with visual impairments or reading difficulties. The device is controlled via a set of key buttons. One central button is used to turn the device on and off, while the other buttons are used to navigate through the options menu and select different options. One of the most interesting aspects of the Victor Reader Stream device is its ability to play content from different sources. The device has an SD card slot, which can be used to store audiobooks, podcasts, and other types of audio content. The device can also be connected to a Wi-Fi network, allowing users to download content directly from the Internet. Another important aspect is that the users can upload text or PDF documents and the device will read the content aloud. This feature is useful for people with visual impairments or reading difficulties, allowing them to access written content in an easy and efficient way. The Victor Reader Stream device can also have a number of other useful features, such as a voice recording function that can be used to record notes and ideas, or an FM tuner that allows users to listen to local radio stations.

Reveal 16i [42] is an innovative digital reading system designed to help visually impaired people access written content in an easy and efficient way. Made by HumanWare, Reveal is composed of two main components: a video camera, which can be placed on top of a book or other document, and a 16-inch LCD screen, which displays text in a clear and easy-to-read manner. Users can adjust the size and contrast of the text to make it as easy to read as possible. It also has a number of useful features, such as a save function that allows users to store documents to access them later. The system can also be connected to a printer, allowing users to print documents read by the device. It can also be used to access content from a computer or a mobile device via an USB cable or via Bluetooth, allowing users to access digital content.

Even if there is a varied range of solutions commercially available, the ones mentioned above being only a small part of them, there is a real need to have a complete system or solution that enables the user to interact directly with other users, as well as with the environment. A highly innovative product that addresses this need is the BrailleNote Touch Plus 32 [43], which is perhaps the most complete system dedicated to the blind. It combines the functionality of an Android tablet with a Braille touch screen to provide an ideal user experience in navigation and communication, or even making graphics like any other person. BrailleNote Touch Plus is a portable device with a weight of about 900 g and a touch screen of 18 cm and a resolution of 1024 × 600 pixels. The Braille keyboard has 32 cells, which allow users to write and read with ease. The device runs on the Android Oreo operating system and has an internal memory of 64 GB. It also has a number of ports, including USB, HDMI, and a headphone jack. The Braille screen is made of piezoelectric cells, which provide increased durability and a pleasant touch. Users can control the device using the touchscreen, physical keys, or voice commands, thus offering maximum flexibility in use. Users can download apps from the Google Play Store and use them through the Braille touchscreen or voice commands. Regarding the hardware configuration, the device benefits from a quad-core processor and 4 GB of RAM, thus providing excellent performance and a fast and smooth experience. Also the BrailleNote Touch Plus benefits from a 21 MP camera and a document scanner that offers OCR recognition functions so that users can convert text from paper into digital format. Bluetooth technology is not missing either, which means that it can be connected to other devices, such as mobile phones or computers, to transfer files or use other useful functions. In addition to the basic functionality of the tablet, the BrailleNote Touch Plus offers a number of functions specific to blind people, such as GPS navigation and reading books in digital format. Users can use the device to navigate the city or travel safely by accessing location information and directions through GPS systems. BrailleNote Touch Plus is compatible with a wide range of file formats, including PDF, DOC, TXT, HTML, and more, so users can read and access content at any time. It should be noted that BrailleNote Touch Plus benefits from its long-lasting battery, which can last for around 12 h of continuous use. The Braille touch screen is located at the bottom of the device and can be used to read and write in Braille, and also to navigate through menus and applications. Voice commands are recognized by the Android operating system and allow users to control the device with voice commands and use applications such as WhatsApp, Skype, Netflix, YouTube, and more. For these applications, the BrailleNote Touch Plus connects to the internet via Wi-Fi. In addition, the device is compatible with other devices, such as smartphones or laptops, and can be used as an external Braille display device.

**Figure 3:**
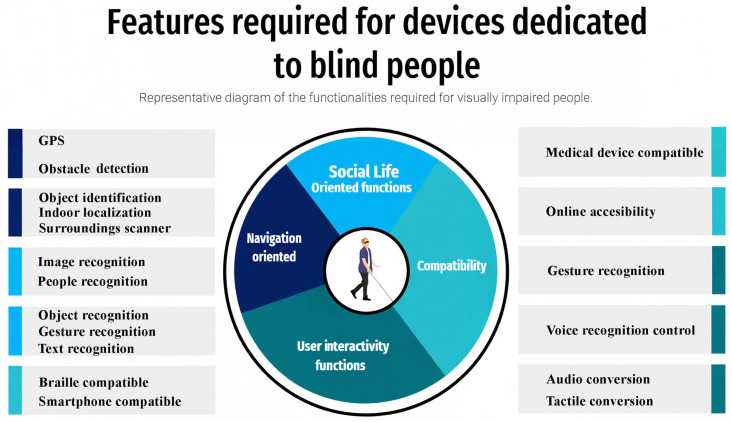
Illustration summarizing the main features of assisting technologies and systems for visually impaired persons.

In addition to these devices, we can also list Canute 360 Premium [44], which is a portable Braille-reading gadget created by the Bristol Braille Technology company. It benefits from a touch screen with nine lines and a total of 360 Braille characters, with an integrated screen reader, which allows the user to access and control the information on the touch screen. Optelec ClearReader+ [45] is a system that allows the user to scan and convert printed text into a legible, easy-to-read format using optical character recognition (OCR). It can also read the scanned text and display the text on an LCD screen. Envision Glasses [46] are smart glasses based on a camera mounted on the frame, that are used in conjunction with the Envision AI mobile application, available for iOS and Android devices. It uses AI technology to recognize objects, texts, and faces, and the user can receive information through voice and tactile feedback. The application can also read texts from books, magazines, and other documents. DOT Watch [47] is the world’s first Braille smartwatch for visually impaired people. It displays the time and other information via a touchscreen with Braille dots, and the user can control the watch using touch gestures. The third generation of DOT Pad [48] is a portable device that uses electromagnetism to power Braille cells, which brings down the price. The users can write and read via Braille keys. DOT Pad is compatible with smartphones and tablets via a Bluetooth connection and can be used to take notes, read documents, and send messages. DOT Pad integrates seamlessly with iOS and iPadOS. A smart glasses system that uses a high-definition camera, a processor, and a screen to improve the vision of visually impaired users is eSight 4 [49]. The system uses augmented reality technology to provide a clear and detailed image of the surrounding environment. BlindShell [50] is a simplified smartphone specially developed for visually impaired users. The device uses an operating system adapted for ease of use through a physical keyboard and voice commands. BlindShell is compatible with apps like WhatsApp, Facebook, and Twitter and allows users to send and receive messages, browse the internet, and make phone calls. The phone includes an SOS button. Based on all these characteristics, Figure 3 summarizes the main features of assisting technologies and systems for visually impaired persons.

**Table 1:** Assistive devices and applications for visually impaired people.

Commercial Name	Manufacturer	Functions	Mode of Use	Supported OS	Cost
Wayband [26]	WearWorks Inc.	Haptic Navigation	Through vibrations from an armband	iOS	Low
Ara [30]	STRAP Technologies, Inc.	Navigation and surroundings narration	Through haptic vibrations and through AI app	Standalone device	medium
WeWalk Smart Cane [31]	WeWALK LTD	Smart detection through audio and haptic means Intelligent voice assistant Navigation instructions	As a smart cane	Standalone device	Low
NVDA [32]	Microsoft	Screen reader	Software	Windows	Free
Be My Eyes [37]	Accessibly Inc.	On-line assistance on demand	Through volunteer service	iOS Android	Free
Aira [35]	Aira Tech Corp.	On-line assistance on demand	Through live agent service	iOS Android	Monthly subscription payment
GoodMaps [36]	GoodMaps Inc.	Navigation	Indoor: geo-referenced images through a camera-based positioning system. Outdoor: through GPS	iOS Android	Free
BlindSquare [27]	MIPsoft	Navigation and surroundings narration	Indoor: through BPS Outdoor: through GPS	iOS	Low
Nearby Explorer [51]	American Printing House for the Blind, Inc.	Navigation	Indoor: through BPS Outdoor:—through GPS	Android iOS	Low
Seeing AI [34]	Microsoft	Text reading Objects and people recognition Barcode scanning	Through AI app	iOS Android	Free
Text to Speech TTS Voice Dream [33]	Applause Group	Text reading	Through mobile app	iOS	Subscription payment
Wayaround [38]	Wayaround	Tag-and-scan	NFC reader	iOS Android	Low
OrCam MyEye 3 Pro [39]	OrCam Technologies	Text and traffic signs readingObjects and people recognitionReal-time navigation	Clips onto the user’s eyeglass frame	Standalone device	High
Victor Reader Stream 3 [41]	HumanWare	Text reading	Media player device	Standalone device	Low
BrailleNote Touch Plus 32 [43]	HumanWare	Tablet with note-taking functions	Through Braille system	Standalone device with Android	High to Very high
Reveal 16i [42]	HumanWare	Magnifying reading text	Through camera	Standalone device	High
Canute 360 Premium [44]	Bristol Braille Technology	Electronic Braille reader	Through Braille system	Standalone device	High
Optelec ClearReader+ [45]	Optelec B.V.	Text reading Text-to-speech function	Through camera	Standalone device	High
Envision Glasses [46]	Envision	Objects and people recognition Real-time navigation	Through camera	Standalone device	High
DOT Watch 2 [47]	DOT Incorp.	Smartwatch	Braille system	Standalone device	Low
DOT Pad [48]	DOT Incorp.	Braille tablet	Braille system	Standalone device	Very high
eSight 4 [49]	eSight Co.	Smart glasses with Augmented Reality	Camera	Standalone device	High to Very high
BlindShell [50]	Matapo A.S.	Simplified smartphone	Through physical keyboard and voice commands	Standalone device	Low to medium

## 3. Perspectives on Visible Light Communications Solutions for Visually Impaired Persons and Artificial Intelligence

### 3.1. Brief Perspectives on Visible Light Communications and Their Potential and Applicability

**Figure 4:**
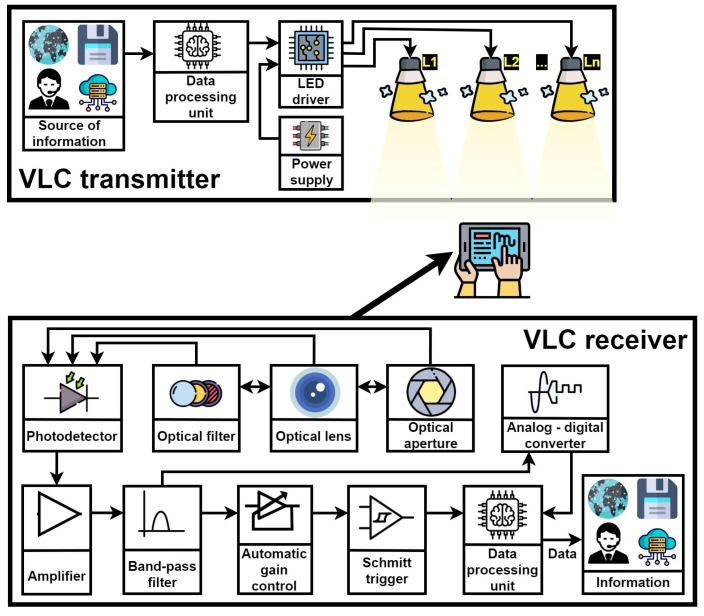
Schematic representation of a visible light communications architecture emphasizing the transmitter and receiver components.

Visible Light Communications (VLC) is a technology which has a high potential to significantly change our lives from many perspectives [52,53,54]. Basically, VLC assumes the use of the light generally used for lighting and/or signaling purposes for wireless data transmission. VLC takes advantage of the current transition from incandescent and fluorescent lighting toward energy-efficient solid-state lighting sources [55,56]. Such lighting devices not only require up to ten times less energy to generate the same amount of light in the visible spectrum, but are also capable of fast switching, enabling them to be used in data transmission purposes. For example, LEDs can have response times that go below one nanosecond, enabling multi-gigabit-per-second data rates [57,58]. Forced by the current trend in developing a climate-neutral society [59], VLC emerged as a wireless communication technology where the data transfer is made without additional energy consumption, as the same light which is used for illumination is used as a carrier for the data as well. It should be emphasized that the current transition toward VLC-based wireless communications is also motivated by the potential for never-before performance. Compared to the classical RF-based wireless communication solutions, which are being developed on a 300 GHz bandwidth, VLC applications bring to the light a 400 THz bandwidth, available without licensing costs. In this context, VLC technology has reached a high maturity level in a relatively short period of time. Thus, current VLC prototypes demonstrated impressive data rates, currently up to tens of gigabits per second [60,61], while having the potential to reach up to 100 Gb/s [62]. Moreover, in addition to these impressive data rates, VLC systems became capable of handling complex networking issues, enabling users’ mobility [63], multi-user connection [64,65], and integration in hybrid RF-VLC networks [66]. Moreover, the intrinsic characteristics of light ensure that VLC data transmissions are contained within a specific area, ensuring better security against eavesdropping. Therefore, based on such remarkable characteristics, it is considered that optical wireless communications will play a major role in future 6G and beyond technologies [67,68]. The compatibility with 6G applications is also ensured by VLC’s capacity to work in conjunction with other wireless communications technologies, providing additional capacity and offloading traffic from congested RF bands, thus ensuring improved overall performance (reliability, resilience, higher data rates, lower latencies, and wider coverage). Consequently, based on solid performance and high potential, commercial VLC systems have become available, proving the transition from high-potential to user-ready applications for various applications, ranging from light-based wireless internet connectivity [69], to music broadcasting [70], to vehicle safety applications [71,72,73]. For a better understanding of the concept and its use, Figure 4 provides a schematic of a VLC prototype, emphasizing its components and their interconnectivity. From a hardware point of view, the VLC transmitter assumes the integration of a data control module installed between the power supply and the LED lighting source. Thus, through a LED driver and a data control unit, the light source is interconnected with a source of information, and in this manner, the light switches on and off, generating a modulated light beam which will carries the data through the free space. At the receiver end, a photoreceiver (i.e., usually a PIN photodiode) converts the incident light into a proportional electrical current, which is then transformed into an electrical signal. For enhanced Signal-to-Noise Ratio (SNR), the VLC receiver can include an optical lens, optical filters, and sometimes an adaptive Field of View (FoV). Next, the electrical signal is processed in various stages until the information becomes available for its user.

**Figure 5:**
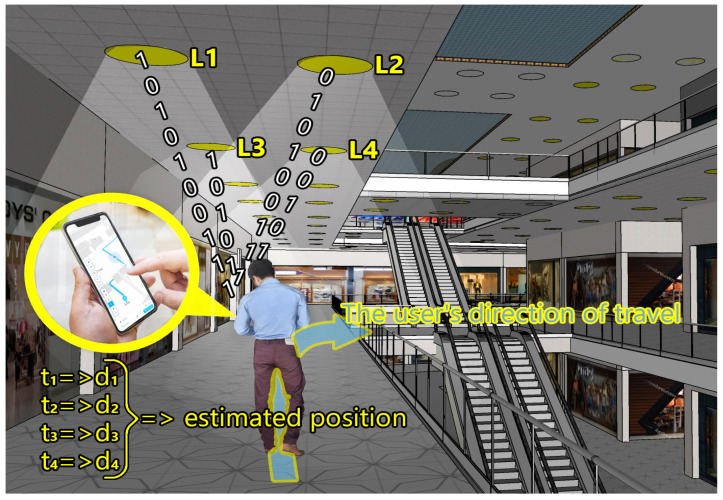
Visible light communications and positioning use case scenario: by using the indoor lighting infrastructure, visible light is used as a carrier for the data, providing high-data-rate wireless communications, whereas, based on signals received from several lighting sources (i.e., L1, L2, L3, L4), measuring the time-of-flight values (i.e., t_1_, t_2_, t_3_, t_4_) and converting them to distance estimations (i.e., d_1_, d_2_, d_3_, d_4_), high precision indoor localization is achieved, enabling indoor guidance services.

In addition to impressive performance and high potential in terms of wireless communications, VLC technology provides additional features, further enhancing its potential. Thus, by using various time-of-flight measurement techniques, VLC can also provide accurate distance measurement between VLC transmitters and VLC receivers, enabling high precision localization. As GPS solutions are inappropriate for indoor applications, this feature becomes a major advantage of VLC. As shown in [74,75,76], VLC technology demonstrated that it is able to provide centimeter-precision localization in 2D and 3D applications. Thus, by providing additional functions in addition to illumination and data transmission, VLC technology can provide extra benefits, while maintaining a rather simple architecture and cost-efficient utilization. Figure 5 shows a VLC-based indoor localization application exemplifying how a person’s localization is determined with respect to the light sources’ location and how user-centered travel path indications are provided.

Finally, due to its high potential for multiple tasks, VLC technology has the potential and the means to be an important component of the ICS concept [19,20,21]. In ICS, VLC explores the visible light spectrum to enable high-speed, low-latency wireless data transfer and precise environmental perception and sensing. These features are available by relying on a ubiquitous lighting infrastructure, becoming a unique candidate for complex environments and applications. In addition to lighting, communications, and accurate indoor positioning, VLC can be used in surface perception [77], obstacle detection [78], and environmental sensing and monitoring [79], enhancing contextual awareness and safety. Furthermore, VLC’s compatibility with the Internet of Things (IoT) concept further multiplies its use case and applicability [80,81], putting the basis for unified connectivity and real-time data communications between different devices and systems. Based on these perspectives, one can see that VLC technology is well-suited for integration in the ICS framework, enabling a unified network that provides the support for complex applications in smart cities, while being well-matched with assistive technologies for persons with various disabilities, including here severely visually impaired and blind people.

In conclusion, the rapid development experienced by VLC technology arouses interest in its use in more and more applications. The benefits made possible by VLC as an assistive technology for visually impaired people can be summarized as follows:

- Real-time indoor navigation: smart glasses or camera devices can provide real-time information about obstacles in their path, helping blind people navigate their surroundings more safely and effectively;
- Accessibility and affordability: many VLC solutions can be implemented on the existing infrastructure, making the technology widely accessible and cost-effective;
- Enhanced privacy: the technology can be implemented discreetly, without invasive procedures;
- Improved surrounding awareness: the VLC can help people to better understand what is around them based on an overview information provided through haptic and audio means.

Nevertheless, the technological improvements lead to a large diversity of characteristics for optoelectronic devices, which increases the implementation and data processing complexity. That calls for AI technologies to better draw the design models, to optimize the transmission link, and to efficiently adapt to the characteristics of the entire network [82]. Even more, the AI technology can bring to the table other benefits, such as:

- object recognition: this could help blind people to have detailed information for easier navigation and interaction with other people;
- Adaptability to user’s preferences: the AI technology can adjust the system to the client’s needs or habits;
- Text and voice interactions: the text-to-speech conversions and vice versa in real-time are possible;
- Improved safety features: the AI can detect hazardous situations and alert the user;
- Early eye-disease detection: in-time detection of eye diseases can help visually challenged persons to take appropriate measures to avoid worsening or losing their eyesight.

### 3.2. Perspectives on Artificial Intelligence

Artificial intelligence has become one of the most transformative technologies of the 21st century, changing the way we live, work, and interact with each other. AI has become a practical tool that enhances efficiency, drives innovation, and improves quality of life thanks to the modern algorithm design innovations. AI encompasses a broad spectrum of technologies, including machine learning, natural language processing, computer vision, and robotics, each contributing to its vast potential and versatility.

AI’s significance extends beyond its ability to automate tasks and processes; it can also analyze complex data sets, uncovering insights and patterns that would be otherwise unattainable, contributing to an increase in the quality of life. Numerous industries, from healthcare and finance to education and entertainment, are experiencing profound changes as a result of this new capability. The power of AI to personalize experiences, optimize operations, improve healthcare services, and predict outcomes is transforming business models and providing new paths for the growth and streaming of new technologies and systems.

Moreover, AI’s contributions extend beyond economic benefits. In healthcare, AI-driven diagnostic tools are enabling earlier and more accurate detection of diseases, leading to better patient outcomes and ultimately saving lives. In the realm of environmental sustainability, AI is aiding in the efficient management of resources and the reduction of environmental impacts by reducing the carbon footprint. The integration of AI into everyday life is also enhancing accessibility, providing support for individuals with disabilities and contributing to their integration in society.

In the next few years, AI will continue to evolve; thus, its ethical implications and potential risks must be carefully managed in order to ensure that its benefits are widely distributed and its adverse effects reduced. Mankind will harness the potential of AI to drive progress and improve the quality of life by understanding its many roles in society. 

## 4. Artificial Intelligence and Early Eye Disease Detection

An important issue that must not be neglected is related to in-time detection of eye disease. If left untreated, eye diseases can lead to severe vision impairment or blindness, with a great risk to affect millions of individuals worldwide. Early detection and diagnosis are crucial for effective treatment and disease-management protocols, potentially preventing the progression of these eye conditions. Traditional diagnostic methods, while effective, often rely on highly trained specialists and can be time-consuming, costly, and inaccessible to patients in remote areas. In recent years, the advent of artificial intelligence has revolutionized various fields of medicine, including ophthalmology. AI-driven approaches, particularly those leveraging deep learning, have shown promise in enhancing the accuracy and efficiency of eye disease detection. These technologies can analyze vast amounts of imaging data, identify patterns that cannot be observed by the human operator, and make diagnostic predictions with a high degree of precision. This section explores the application of AI in the detection of eye diseases focusing on Keratoconus (KCN) detection. In this section, we review the current state of AI technologies in ophthalmology, highlighting key advancements, methodologies, and the integration of AI systems in clinical environments. Additionally, we address the challenges and limitations associated with AI implementation, such as data quality, algorithm transparency, and the need for objective evaluation criteria.

Keratoconus is a progressive non-inflammatory disease characterized by thinning of the cornea and deformation of the inner or outer corneal surface [83]. The progressive deformation of the cornea can lead to visual acuity deficiency conditions such as irregular astigmatism, Higher-Order Aberrations (HOAs), progressive myopia, and corneal thinning [84]. Detection of keratoconus is typically straightforward at severe stages of the disease; however, its detection could be challenging at earlier stages, even for experienced ophthalmologists [85]. The diagnosis and management of keratoconus have been improved substantially over the past few decades, partly due to advances in corneal topography and tomography imaging [86] which have allowed more accurate and reliable measurement of corneal curvature, thickness, and elevation. For instance, Scheimpflug technology and Optical Coherence Tomography (OCT) imaging systems have provided more detailed images of the cornea [87].

Even with the development of corneal imaging systems, keratoconus diagnosis, particularly at earlier stages of the disease, is clinically challenging, yet critical, as progressive keratoconus may impact corneal stroma, leading to an increased likelihood of future invasive refractive surgery. As these diagnostic systems have become more advanced, computational models play a significant role in detecting keratoconus from corneal images. Conventional machine learning (i.e., ML) has been widely employed for keratoconus detection [88]. Such approaches have used graph theory [89,90], fast active contour and polynomial fitting [91], Canny edge detection [92], Gaussian mixture models [93], and Hough transformation combined with Kalman filtering [94]. 

Recently, a subset of artificial intelligence algorithms called Convolutional Neural Networks (CNN) has received interest in ophthalmology and have been widely applied to retinal images [94,95,96]. However, their application in the corneal area has not received as much attention as for the retina. Nevertheless, as CNN models inherently recognize patterns (e.g., keratoconus-induced signs in the topography images) very well, they have become an excellent candidate for detecting various ocular conditions from imaging data [97]. 

Several scientific contributions have studied the capability of deep CNN models to detect keratoconus-based corneal maps collected based on Scheimpflug or OCT technologies [98,99,100]. Some of them have used a hybrid perspective that includes a classical ML approach on features extracted via a Deep Learning (DL) technique [101]. Moreover, our previous study used simulated corneal images for keratoconus detection [100], establishing a foundation for future research [101,102]. Therefore, in this paper, the development of a deep CNN model to detect keratoconus based on clinical OCT-based corneal images captured by the CASIA instrument [103,104] is presented. It has been shown that the proposed model detects suspected keratoconus with a high degree of accuracy, making it a potential candidate for clinical applications.

**Figure 6:**
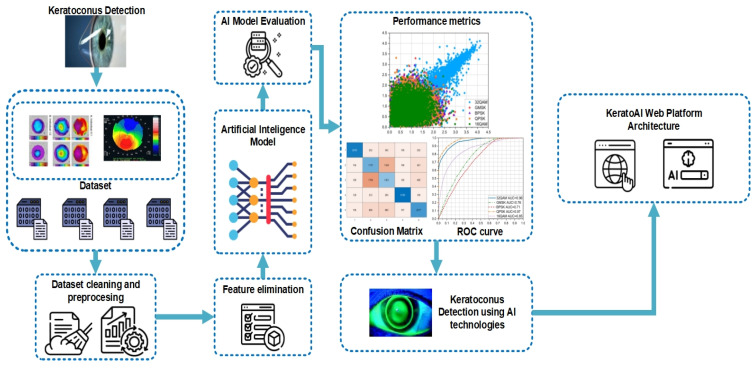
Workflow of eye disease detection using AI algorithms.

Figure 6 presents the main workflow of eye disease detection using AI algorithms that includes dataset preprocessing, feature elimination operations, AI model development (training, validation, and testing), and finally integration and deployment.

The advantages of using AI models are becoming increasingly evident [105]. The performance level of many state-of-the-art AI models (i.e., DL models) can be extremely high, and the demand for computing resources has gradually reduced. Supervised learning is a method that uses artificial intelligence for classification tasks, where the data labels are known, compared to unsupervised techniques, where the labels are not included in the training process. There are basically four methods of classical ML: supervised, unsupervised, semi-supervised, and reinforcement learning. The most used types of unsupervised learning are clustering, association, and anomaly detection [106].

Until recently, in many classical ML approaches, features were manually extracted, but now, with the help of DL, the features are automatically learned from data. DL is a subset of ML algorithms that can learn patterns similarly to the human brain. However, DL provides a black box model, as the input processes are highly involved and challenging to be tracked in detail. In this section, we review some of the current AI models presented in the scientific literature for keratoconus detection.

Rozema et al. [107] developed and validated a stochastic eye model for keratoconus development. The SyntEyes KTC archetype was designed for researchers who do not have access to real eye data. Therefore, 145 right-sided eyes were collected and examined by Scheimpflug tomography and eye biometrics, being affected by keratoconus. The data were processed using Principal Component Analysis (PCA) to reduce the number of parameters that make up the images and then passed through a Gaussian multivariate fit that produces the stochastic model for keratoconus. In addition, a filter is connected to the output to eliminate unwanted patterns. Finally, the SyntEyes KTC is compared to a healthy eye model that uses non-corneal biometry to describe the development of keratoconus, especially in its early stages. In this way, it is possible to generate an unlimited and random number of biometric sets with the same statistical and epidemiological properties as the data taken from reality.

Lavric et al. [102] implemented and tested, for the first time, the CNN-based KeratoDetect algorithm that is able to extract and learn the characteristics of keratoconus-affected eyes. The most common way to diagnose the disease is by corneal topography, an interpretation made by specialists in ophthalmology. The images taken by topography represent the algorithm’s input data that uses colored scales to identify the cornea’s curvature. The topometric method displays colored maps in absolute and standardized scales, leading to a total cornea image, but KeratoDetect uses synthetic computer-generated data. KeratoDetect, in the learning process associated with the CNN decomposes the image at the pixel level and arranges it in the form of a matrix. The data are passed through various layers, such as convolutional kernel filters, pooling layers, and cognitively connected (FC) layers. Then, the classification is done in the output layer by the Softmax function by using probabilistic values. The implemented algorithm processes typical corneal topographies and classifies them into two categories, including the detection of keratoconus-specific behavior. The results led to an accuracy of 99.33% on generic generated data and can aid ophthalmologists in disclosing the disease as quickly as possible with a very low diagnostic error. 

CorneaNet is a neuronal network [108] that uses healthy and ill eyes with three corneal layers, i.e., epithelium, Bowman’s layer, and stroma, in a DL approach. The advantage of the developed algorithm is that it can also be considered for other diseases, not only keratoconus. The obtained accuracy is around 99.56%, but the dimension of the dataset is relatively low, with only 72 healthy eyes and 70 keratoconus eyes.

In [107], a hybrid diagnostic system of incipient keratoconus was developed. This system is based on a mathematical approach, a feedforward neural network (FFNN), and a neuronal architecture (Grossberg–Runge Kutta) which consists of a supervised learning process and coordinates the weights between connections to a classification category output. Also, the algorithm proposed by the authors reduced the simulation time by 70% compared to the standard ML algorithms, and the accuracy was 96.56%. The method used only corneal elevation and thickness dimensions for a total of 851 subjects, as a small data sample represented the training set.

Kamiya et al. [109] realized a study on the accuracy of diagnosis and identification of the keratoconus stage with DL, where the input data were color-coded maps measured with an AS-OCT device. The CNN used was the ResNet-18 network, and the total number of eyes used in this study was 304. The recorded overall accuracy was around 0.991 for the small dataset used.

Kuo et al. [108] developed three types of DL algorithms that were evaluated for detecting keratoconus and its validation with specific methods. With the topographic method, 354 images were acquired, belonging to three eye categories (170 keratoconus, 28 subclinical keratoconus, and 156 healthy). The sensitivity and specificity values for the three models are 0.90 for CNN, and the AUC reaches 0.995 for the ResNet152 algorithm. The essential characteristics were obtained from the pixel and the thermographic map for prediction using the VGG16 architecture model by the gradient difference of the topographic map. The three CNN algorithms’ accuracies were 0.931 for VGG16, 0.931 for INceptionV3, and 0.958 for ResNet152. The DL models gave a high accuracy for detecting keratoconus using topographic images of the cornea. The method mentioned in their study showed that the model focuses on the appropriate region for diagnosis and clinical interest, stating that DL and AI are highly valuable resources for diagnosis in ophthalmology.

The Issarti et al. [110] study included 812 subjects divided into two group categories: healthy (304 eyes) and keratoconus (508 eyes). The comparisons were realized between the logistic index for keratoconus (Logik), Belin/Ambrosio Display Deviation (BAD_D), and the Pentacam Topographical Keratoconus Classification (TKC). The authors determined the overall accuracy of the Logik algorithm at around 99.9%. A Feed Forward Neural Network (FFNN) algorithm was used, followed by a Moving Average Filter (MAF) to obtain a platform that independently classifies keratoconus identification and eye grouping according to its degree. MAF is a filter that creates a series of subset averages from the complete training dataset.

An automatic classification system has been presented in [111] in order to distinguish healthy eyes from those affected by keratoconus. The study involved 121 participants, and the images were acquired using the Scheimpflug method and UHR-OCT (Ultra-High-Resolution Optical Coherence Tomography) devices. A neural network was used to train a model based on corneal patterns in order to distinguish suspect keratoconus eyes from healthy ones. The Fisher system was used to highlight and rank each individual trait according to its importance. The obtained area-under-the-curve parameter is about 0.93 for a two-fold classification problem (healthy and keratoconus eyes).

Abdelmotaal et al. [112] evaluated the use of DL to classify high-performance images of color-coded corneal maps obtained using Pentacam equipment. The CNN performance was assessed using standard metrics, detailed error analysis, and network-enabled maps. The registered images were corneal front elevation, back elevation, thickness, and front sagittal curvature (for map-selectable display images) with different parameters for a total number of 3218 eyes. The architecture classifies the eyes into three groups: keratoconus, subclinical keratoconus, and healthy. The evaluation of the results led to an accuracy of 95.8% of the dataset used for the test objectives.

Zéboulon P. et al. [113] proposed a CNN network that can process and classify the corneal topography raw data obtained from Orbscan examinations for the first time. The study analyzed three classes of healthy eyes, keratoconus, and history of refractive surgery, and received an overall accuracy of 99.3%. Still, one disadvantage of the model is the lack of generalization of the dataset obtained using only the equipment settings.

On the other hand, Ali H. Al-Timemy et al. [114] presented the design of a hybrid DL construct for detecting keratoconus from corneal maps that uses a feature-fusion technique that is next classified by using a support vector ML algorithm. The research idea was to use an independent dataset in order to evaluate the performance level of the keratoconus detection algorithm that was trained on 3794 corneal images. The obtained accuracy was 92% in distinguishing normal from keratoconus eyes and 68.7% when also detecting suspect keratoconus eyes. In Table 2, we summarize the performance level or main AI algorithms used for keratoconus detection.

Despite the clear advantages, the implementation of AI in ophthalmology is not without challenges. Ensuring the robustness and generalizability of AI models across diverse populations and clinical environments is essential. Additionally, addressing issues related to data privacy, algorithm transparency, and gaining regulatory approval will be crucial for the widespread adoption and trust in these technologies. In conclusion, AI algorithms hold immense potential to revolutionize eye disease detection, offering faster, more accurate, and accessible diagnostic solutions. By continuing to advance these technologies and overcoming existing challenges, AI can play an important role in improving eye health outcomes and enhancing the quality of life for millions of individuals worldwide.

## 5. State of the Art in Visible Light Communications Solutions for Visually Impaired Persons

As debated in Section 3, VLC technology has unique features and high potential, being a suitable candidate for a multitude of applications. Specifically, based on unique characteristics, VLC technology has a very high potential to be used in blind and visually impaired persons’ assistance. Nevertheless, despite a remarkable potential, this domain seems to be insufficiently explored. Thus, the following section aims to put the spotlight on the existing efforts oriented toward the use of VLC in visually impaired persons’ assistance, providing a historical evolution of the concept.

Before moving forward, it should be remembered that the development of VLC technology began in the early 2000s, with a relatively slow start. Due to specific issues and numerous challenges, the development was rather slow in the first years, with relatively limited progress. Next, after 2005, the pace of development increased, and has continuously accelerated up until today. Then again, the progress achieved has been little applied in blind persons’ assistance applications, pointing out that the technological progress should be made accessible for vulnerable users.

In this context, among the first works which considered the use of light sources in visually impaired persons’ localization are [116,117,118]. In this case, fluorescent light sources are used to transmit their position information toward a mobile optical receiver. By joining the information from more light sources, the system precision is improved [116]. Moreover, for better confidence and higher precision, the authors also simulate the use of Bluetooth and RFID technologies for user location determination [117]. The practical implementation of the VLC system is experimentally evaluated in [118] with human users. So, based on an optical receiver carried by the user, the information from several available light sources is put together and the user location is estimated. Next, this information is transmitted through Bluetooth from the optical receiver to a PDA, which provides the information to the user as a voice message. Although the proposed architecture has room for improvements, experimental results showed in a practical platform that the average distance error could reach as low as 10 cm. Thus, these works emphasized that fixed light sources can be used to provide location beacons for visually impaired persons.

In [119], the authors have analyzed the problems and needs that people with visual impairments have when traveling unfamiliar routes, focusing on signalized intersections. Based on information collected through studies and questionnaires, a system dedicated to these problems was designed and implemented. The authors demonstrated that the proposed solution can guide a person with disabilities in the process of safely crossing an intersection. The authors have developed two types of receivers, one that can be placed in the chest pocket, and the second as a portable handheld device. The mechanisms of the receivers are identical, so subjects can hear phonetic information from the headphones when the receiver lens picks up the signal from pedestrian or road traffic. When the direction is lost, they receive a noise that indicates that they do not have a viable trajectory, understanding that they must wait for appropriate guidance through the VLC system. The system has been tested in various conditions, in indoor and outdoor environments, demonstrating its benefits. In terms of range, the system can cover a 20-m distance, being suitable for outdoor applications. 

A different VLC architecture for blind individuals’ assistance is proposed in [120]. This work analyzes the use of PPM and OFDM modulation, while also emphasizing the importance of privacy and data cryptography in such applications. Although the experimental evaluation is rather basic, preliminary results indicate communication ranges of several meters, confirming the potential in indoor applications.

A complex VLC assistance solution is proposed and modeled in [121]. In this case, an information display is considered as a VLC transmitter, broadcasting navigation signals for users within its vicinity. The concept considers multi-channel communications and analyzes the impact of other artificial lighting sources on the system’s performance. Additionally, as the system could be installed in areas where signal reception could be blocked by other users, the authors analyze the impact of error-correcting methods. Simulation results indicate that the concept is suitable for ranges up to a few meters, showing that the impact of optical interference can be partially mitigated with the help of error-correcting codes.

A performant VLC-based navigation solution for blind individuals is proposed and experimentally validated in [122]. Similarly to previous concepts, this one envisions indoor navigation based on VLC-transmitted mapping information which is then transformed into voice messages. Nevertheless, for improved performance, a smartphone’s geomagnetic sensor is used to determine the users’ orientation and travel direction. The prototype has been implemented in a building and tested with real blind users. During the tests, the system proved to be suitable to assist the users in traveling to a certain point within a test course which involved several direction changes. On the other hand, in certain moments, the users felt uneasy about the direction they followed, pointing out that continuous feedback concerning the route would be helpful. Considering the importance of identity authentication in various applications of daily lives, the authors of [123] proposed visible light fuzzy timing password validation suitable for disabled and visually impaired persons. Analytical evaluations confirm that due to VLC particularities, an intruder is less likely to capture elements of the data packets. 

The authors of [124] propose an optical wireless communications solution aimed to assist pedestrians with visual impairment in danger or obstacle perception. For this purpose, an optical communication system consisting of an illuminated bollard transmitter and a receiver terminal is proposed. The system uses 4PPM modulation and provides a data rate of 125 kbps. The evaluation experiment with visually impaired people showed that the best distance to announce a notification of danger is approximately 2 m. The luminescent color and modulation format of the bollards were discussed, and prototype models for 2 m visible-light communication were demonstrated. As part of the preliminary tests, the concept was only evaluated in a dark room to design a pedestrian support system at night. The concept is further enhanced and evaluated in [125]. At this stage, the VLC receiver is designed for a person’s neck, arm, and white cane. So, as the person is approaching a bollard having an incorporated VLC transmitter, information concerning a potential danger is received through VLC. Thus, the presence of stairs, or a sidewalk or of a certain point of interest can be identified. The benefits of the concept have been confirmed experimentally by several blind persons.

Tactile pavings help visually impaired persons by pointing the pathways and escalators, acting as auxiliary navigation systems. As the 10-m distance accuracy of GPS systems is unsuitable for the visually impaired, the authors of [126] proposed a new method using visible light communications transmission from tactile pavings. This method provides higher accuracy due to the short distance between the transmitter and receiver. In this work, VLC is used to transmit information through flashing lights, which in this case is more precise due to light’s linearity. To mitigate interference from signals coming from neighboring tactile pavings, a Code Division Multiple Access (CDMA) is used. The prototype’s experimental evaluation confirmed the high precision.

To address the challenges that visually impaired persons have to face when navigating unfamiliar public places, the authors of [127] propose a user-friendly and comfortable audio guidance system. The concept uses VLC technology, geomagnetic sensors, and obstacle detection to address positioning and navigation problems, and voice commands and beep sounds for guidance. The system uses the indoor LED lights to transmit location information and an enhanced version of Dijkstra’s algorithm to determine the best path, considering search time and distance. For improved traveling experience, geomagnetic sensors are used for directional guidance, while sonar sensors are used for obstacle detection and collision prevention. Although rather well designed, the concept does not make the transition toward a working prototype. 

The authors of [128] acknowledge the potential of VLC and VLP in blind persons’ assistance and provide a fundamental analysis concerning the accuracy of VLP. Their study focuses on communication and localization using the indoor lighting system and indicates BER performance in the 10^−6^ limit, a rather high accuracy in user position estimation. On the downside, this work points out the negative effects introduced by reflections on the wall. From this reason, it is emphasized that the corner of the room becomes the area with the lowest SNR.

A practical solution for blind individuals’ assistance is proposed and evaluated in [129]. In this case, the authors consider an illuminated sign acting as a VLC transmitter, and a VLC receiver integrated in a pair of smart glasses. The proposed concept has been implemented as a proof-of-concept prototype and experimentally evaluated. For simplicity, the system employed analog transmission of audio signals. The experimental results showed a communication range of up to 2 m and an acceptable audio quality. Another positive aspect of this work comes from the low cost of the receiver, which was less than 10 USD, thus enabling wide accessibility for the system.

The authors of [130] propose the implementation of a light-tracking system for visually impaired people. This system facilitates the movement of blind people in the orientation process and maintains mobility under indoor conditions. The transmitter is developed as LEDs which are integrated within the floor, marking the travel path. The VLC receiver is designed in the form of a blind stick and mainly consists of an optical receiver, a buzzer, and a timer. Light measurements are made based on acrylic and color filters which are used to increase the accuracy of the detection, while also taking into account the angle or the conditions in which the fluorescent lights are on or off. This approach showed that the system can transmit audio signals at a frequency of 3000 Hz. The results demonstrated the viability of the concept in guiding blind people though a VLC-based blind stick. Another VLC-based blind stick is proposed in [131]. The paper proposes an indoor navigation system for the visually impaired where VLC is used to obtain audio information. Data transmission occurs through VLC after identifying the person who has entered the room. The efficiency of the Li-Fi transmitter and Li-Fi receiver is investigated by testing it at different transmitter—receiver ranges. The Li-Fi module data were obtained by using the light intensity measurement and the sound meter to measure the volume of the command. The ultrasonic sensor was tested with random distances to check the function of distance calculation, confirming its proper functionality. Thus, obstacles are located, and different sound frequencies are generated, depending on the distance from the obstacle.

To improve indoor navigation for blind people, Li-Fi technology was also employed in [132]. For extra support in guiding visually impaired persons in unfamiliar environments, the system includes an ultrasonic sensor for obstacle detection, and an accelerometer which is used to determine the user’s angular velocity. Although the system’s implementation is not at a level which is suitable for real applications, this work emphasizes the VLC potential in such applications. The use of VLC in such applications is also debated in [133,134,135,136,137,138]. In these cases, the benefits of VLC utilization are emphasized, and basic demonstrations are provided. Nevertheless, the hardware implementations remain at a rather basic level, without providing the possibility for real-life use. 

**Figure 7:**
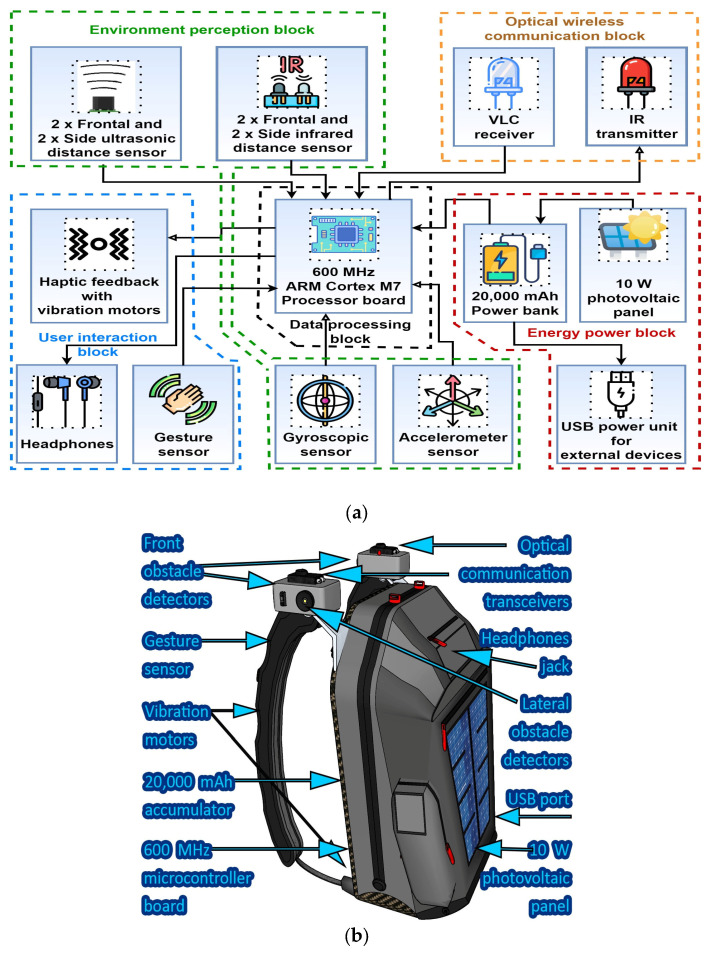
Visible light communication-based smart backpack for blind and severely visually impaired persons’ assistance: (a) Schematic representation; (**b**) 3D design [139].

As far as we know, one the most complex and advanced VLC-based blind persons’ assistance solutions is presented in [139,140,141,142]. This concept consists of a data transmission component which has been integrated as part of the indoor lighting component, and a VLC mobile device which has been integrated as part of a smart backpack. For improved versatility, the smart backpack also integrates a photovoltaic panel and a battery for energy storage, enabling the system to be energy-independent. In terms of data communications, the backpack integrates two optical receivers which enable data reception from the indoor lighting system. The received light signals are processed by the VLC receiver until the information is recovered. Next, the received digital data are translated into user-relevant information which is delivered for the user as audio messages. Moreover, for situations in which the blind person requires hearing for other purposes, the information can be delivered by haptic means through several vibration motors located in different zones. Then, for situations in which the user requires certain information in a discrete manner, the backpack includes a gesture sensor which is able to translate certain gestures into specific requests. Next, the user’s request is transmitted toward the indoor lighting system through IR communication. Additionally, to enable the user to travel through unfamiliar environments, the smart backpack includes several distance sensors which scan its surroundings. These sensors are able to detect obstacles around the user and provide an accurate mapping of the area delivered to the user by haptic means. Figure 7 illustrates the schematic of the VLC prototype and its experimental testing. The proposed prototype has been experimentally validated in various conditions and situations relevant for real life applications. The experimental results demonstrated that the VLC component provides highly reliable communications, BER which are lower than 10^−7^, and wide area coverage which extends up to more than 3.4 m around each light source. Moreover, the experimental evaluation proved that the system enables the user to locate obstacles or other persons with a very high accuracy, preventing the user from bumping into them. Additionally, the system proved to be capable of enabling the user to maintain a straight line when walking, and to significantly improve user confidence and traveling speed. Last but not least, considering the importance of energy efficiency, the VLC transmitter lighting component is also compatible with light dimming, being able to constantly adapt the illumination level in accordance to the working space illumination level [141]. Thus, in conditions in which natural light is available, the VLC illumination level is proportionally adjusted, while maintaining the active data communication link [142]. From this perspective, the system demonstrated its capacity to adjust light intensity in the 1–99% range.

## 6. Discussions and Future Perspectives Regarding the Use of Visible Light Communications and Artificial Intelligence Technologies in Visually Impaired Persons Assistance Systems

### 6.1. Perspectives on Visible Light Communications and Artificial Intelligence Integration

As previously outlined, both AI and VLC technologies have the potential to address the needs of vulnerable users, such as those with visual impairments. In light of these considerations, it is recommended that new and emerging technologies be developed in accordance with a policy of “no man left behind.” Consequently, as new technologies emerge, it is imperative that the needs of vulnerable users are considered and that applications are developed to assist them.

It can be reasonably argued that the integration of AI and VLC could represent a significant advancement in the improvement of quality of life and the assurance of independence. Consequently, AI is capable of providing capabilities in environmental perception and in user-centered assistance, which will enable an accurate and user-adapted translation of visual information into formats that are easily understood by visually impaired individuals. In contrast, VLC offers a widely available wireless communications network that is capable of delivering unprecedented data rates and extremely low latencies, as well as highly precise indoor navigation. The combined potential of the two technologies is enhanced, enabling a superior and more tailored response to specific user needs.

Enhanced navigation solutions: The integration of AI and VLC can facilitate the development of enhanced navigation solutions. AI algorithms can deliver context-aware guidance in real time, providing relevant information adapted to the user’s needs and behavior. VLC can further enhance the user experience through its high precision localization and constantly updated information, delivered through a widely available lighting network. This ensures continuous connectivity and rapid access to online information and resources.

Obstacle detection and avoidance: It is evident that the application of artificial intelligence in the perception and analysis of environments can enhance obstacle detection capabilities. This is achieved by analyzing information from a multitude of sensors, including ultrasonic and infrared sensors, and by envisaging potential hazards. In this context, machine learning (ML) models can be trained to identify specific scenarios, analyze vast amounts of information provided by environment sensors, and to respond to complex situations. In such scenarios, visible light communication (VLC) can be used in conjunction with AI to provide real-time updates regarding obstacles or points of interest, dynamically adjusting the guidance provided to the user.

Interaction with the environment and with smart devices: In the context of the transition towards smart places and internet-connected objects (IoT devices), the ubiquitous character of VLC can ensure the support for seamless connectivity between the smart devices, providing the backbone for an ultra-reliable communication network. In such circumstances, AI will be responsible for handling the received information, determining its relevance with respect to the user’s needs, and delivering it in a suitable manner appropriate for a visual impaired user. Furthermore, as the user may be in a state of continuous movement, the VLC capacity to provide location updates will ensure that the received information is relevant for the user.

### 6.2. Joining Forces: Mutual Benefits and Technological Challanges in Visible Light Communications and Artificial Intelligence Integration

AI and VLC are technologies with unique specific characteristics. Nevertheless, their joint potential is even greater, as each of the two can benefit from the advantages of the other. The most straightforward benefit for VLC is given by the enhanced data interpretation which can be provided through AI. Thus, as VLC is subject to multiple types of interference, machine learning algorithms can be used to process and identify signal patterns that could be neglected by human-developed systems. Consequently, the development of improved signal processing techniques and enhanced filtering based on AI techniques could be undertaken, thereby enhancing the capability to cope with low-SNR signals. This integration would result in an improvement in overall performance.

As shown in [142], VLC solutions are beginning to be used in energy-efficient lighting applications. This concept envisions that the VLC duty cycle, and in turn, the optical power and the energy consumption are adapted in accordance with the overall space illumination level, user presence, user’s activity, and user preference. In [142], it has been demonstrated that a VLC system can monitor the illumination level at the user level, and constantly optimize the luminous output, while maintaining the communication link. The integration of AI functions represents a significant advancement in this concept, as AI algorithms can be employed to analyze user activity and adjust illumination levels in order to optimize energy consumption, while simultaneously addressing the user’s needs. Such an approach can provide an optimal balance regarding the illumination level between the needs of those who can see and those who are visually impaired.

Additionally, AI can be employed to enhance the positioning accuracy of VLC systems. AI has the capacity to combine the benefits of current VLC-based localization methods (for example, the combination of TDoA with fingerprinting techniques) and to further improve localization accuracy based on machine learning algorithms and deep learning techniques, as well as on the ability to analyze large datasets.

AI can also provide VLC with predictive maintenance based on early-stage fault detection. From this perspective, AI can predict potential failures in VLC systems by analyzing usage patterns and performance metrics such as illumination level decrease, enabling proactive maintenance, reducing downtime, and also contributing to energy consumption reduction.

On the other hand, AI can also benefit from VLC from several points of view. Most importantly, VLC can provide AI with real-time data access. As AI applications depend, in many cases, on real-time data access, whereas in some cases, AI relies on edge-computing data processing, the capability of VLC to deliver high-speed, low-latency communication is crucial for AI applications, having the potential to significantly improve its performance. Moreover, VLC’s large bandwidth can enable AI to have access to data-intensive applications, enabling it to transmit complex datasets and signals, including high-resolution images and video streams. Thus, AI algorithms can analyze large amounts of data and can offer in-depth feedback and assistance. As previously mentioned, VLC localization accuracy can be improved based on AI algorithms. Nevertheless, accurate localization information can also improve the consistency of the navigation aid delivered to the visually impaired persons. Additionally, VLC’s inherent security provides AI solutions with protection for the sensitive data. As data becomes a valuable resource, the VLC capability to ensure data privacy becomes very important.

The combination of AI and VLC provides synergistic benefits as well. In particular, the context-aware assistance provided by the two will be superior, as AI will gain access to a superior understanding of the context based on the VLC-received data, thus being able to access data which will enable it to identify objects, situations, or hazards in real time, providing users with superior awareness, delivered in a timely manner. 

Additionally, the combination of the two will enable real-time interoperability with other AI-driven applications and systems, establishing the basis of a smart environment capable of assisting vulnerable users. Hence, AI will act as the brain, being responsible for data analyses and decision making, whereas VLC will serve as a communication backbone which will ensure the interoperability between different assistive technologies. Also important, the AI-driven VLC architecture will benefit from more efficient resource management. Hence, AI can handle the optimal utilization of VLC resources by intelligently dealing with bandwidth allocation, prioritizing critical communications, and ensuring a wise use of existing infrastructure. Additionally, AI can optimally handle issues such as user mobility, and handling multiple users. Therefore, by combining the brain of AI and the strengths of VLC, one can expect to create a resilient, effective, user-friendly solution capable of assisting visually impaired persons, providing enhanced independence and better quality of life.

On the other hand, some technological issues emerge from the combination of the two technologies. One of the most important ones is related to the high computational power required by AI operation. In turn, the higher computation power can impose edge computing use, and thus generate higher costs. Alternatively, insufficient computing power can increase the system’s latency, potentially affecting the reliability. Another possible disadvantage comes from an initial higher deployment cost. Although VLC is developing on top of a pre-existing and widely available lighting infrastructure, as both VLC and AI are relatively new technologies, the initial costs can be rather high, postponing deployment of such systems. From this perspective, VLC currently increases the cost with its hardware complexity, whereas AI increases the cost through its resource-intensive specificity associated with AI algorithms, large data sets, and complex training. 

Nevertheless, the main disadvantages are related to AI integration in general, while emphasizing privacy concerns and generalization concerns. By harnessing massive data sets and sophisticated algorithms to perform tasks with unprecedented efficiency and accuracy, AI has revolutionized many sectors. However, the rapid integration of AI technologies has brought significant privacy concerns to the surface. These concerns primarily revolve around the collection, use, and protection of personal data. The extensive datasets required for AI systems often contain sensitive personal, behavioral, and biometric information, raising critical questions about informed consent and data protection for visually impaired systems. In addition, the security of these large datasets is important, as they are attractive targets for cyber-attacks and risk exposing sensitive information. Proper storage and restricted access are essential to mitigate such risks.

Anonymization and de-identification of data are essential strategies to protect individual identities. Transparency of AI algorithms is another critical issue, as the complex and black-box nature of these systems makes it difficult for individuals to understand how their data are being used, and makes accountability difficult. Ethically, it is imperative to ensure that individuals are fully informed about how their data are being used. In machine learning, the challenges of overfitting and underfitting are central to a model’s ability to generalize from training data to unseen data. Training methods such as cross-validation and regular performance monitoring can further improve the overall accuracy. Understanding and addressing these issues is essential to improving the reliability and applicability of machine learning models in real-world scenarios, offering support to visually impaired individuals.

### 6.3. Development Framework

Having considered the potential applications of AI and VLC integration in assisting visually impaired individuals in the previous section, we will conclude this work by proposing a development framework for AI-driven VLC solutions designed to assist blind persons. In light of the aforementioned considerations, a number of research directions have been identified and briefly discussed within the context of this framework.

**Figure 8:**
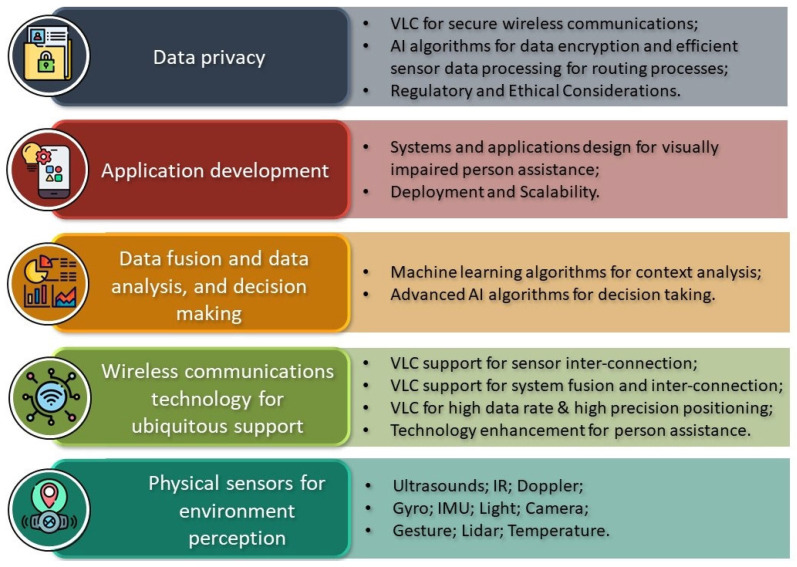
AI-driven VLC assistance solution development framework.

The proposed framework is illustrated in Figure 8 and relies on physical sensors and systems which are required to collect environment information. These sensors have the ability to interact with and monitor the soundings, offering mapping capabilities in real time conditions.

The next layer consists of a wireless communications data transport section; in particular, a VLC is used to connect data collected from various sensors, systems, and components. By considering the perspectives related to the development of the Internet of Things (IoT), it can be anticipated that the data used in the assistance of visually impaired individuals will not be collected by environmental perception sensors carried by users, but will result from a complex data fusion process which uses data collected by portable devices, data from the building infrastructure, and information provided by various devices connected within an IoT network distributed across the premises of a city.

After considering the VLC potential for wide area distribution, it is straightforward to consider it as the backbone infrastructure providing the support for the connectivity of these devices. At the next level, machine learning and AI algorithms should be applied for data fusion, data analysis, and decision making, offering reliable support. Specific to AI-driven systems, such solutions will constantly evolve, becoming capable of understanding and to coping with the users’ needs. Then, once the basis is settled, new assistance applications should be developed while focusing on a user-centered design. Also at this level, deployment and scalability issues should be considered. From this perspective, the capability to develop VLC on a lighting infrastructure which is already available in public and private spaces simulates rapid and efficient deployment, while also contributing to better cost-efficiency. It should be emphasized here that the development of such assistance solutions should focus on producing cost-effective solutions which are reachable for a wide range of users, facilitating widespread adoption. From a data privacy point of view, the involved parties (i.e., users, stakeholders, and governmental institutions) should establish clear guidelines for data privacy and security, ensuring the protection of personal information and its legitimate use. Additionally, as we are discussing applications somehow connected to persons’ lives, ethical use of AI applications should be promoted. More precisely, mechanisms that ensure fairness and responsibility of AI decision-making should carefully be considered and integrated in the framework.

Finally, the framework should facilitate a user-friendly interaction with the system by providing non-intrusive feedback to the user while offering customizable choices in the preferred interaction mode, which is fully reconfigurable in consideration of the user experience.

## 7. Conclusions

In the context in which a representative percentage of the world population is affected by visual impairment, this article focused on the perspectives related to the use of two emerging technologies in blind persons’ assistance applications. Thus, as there is a relatively large amount of work focused on this topic, as well as several comprehensive survey articles which focus on the development of blind individuals’ assistance solutions, this article was oriented toward the use of AI applications and of VLC technology. From this perspective, as far as we know, this is the first comprehensive work which addresses the integration of AI and VLC in such applications. 

It is important to note that both AI and VLC technologies have made significant progress in recent years. Their future development is also expected to be remarkable. Consequently, the potential for these technologies to be used in visually impaired assistance solutions is considerable.

Before moving toward future systems, the first part of this work made a summary of existing commercial solutions, pointing out their features. Next, VLC and AI technologies were briefly presented, emphasizing their compatibility with visually impaired persons’ assistance. As early detection of eye problems represents an important desire which can ensure medical treatment and severe visual impairment prevention, this work addressed the use of AI techniques in early eye disease detection, emphasizing the high accuracy of AI-based techniques. Next, considering the high potential of VLC to provide the support for blind users’ assistance applications, the article provided a summary of existing works focused on the development of such applications. Finally, this work has provided some discussion concerning the roadmap for AI and VLC integration in visually impaired individuals’ assistance. Additionally, a comprehensive AI-driven VLC assistance solution development framework is proposed and discussed in detail. This framework will serve as the foundation for next-generation assistance systems.

In conclusion, this article aims to point out the high perspectives associated with the use of AI and VLC in blind persons’ assistance and to stimulate the research efforts toward this direction.

## Figures and Tables

**Table 2:** Review of keratoconus detection using advanced AI algorithms.

Study	Images	Classes	Patients	Measurement Equipment	AI Algorithm	* Acc (%) AUC
Lavric A. [102]	3000	2	NA	NA	CNN	99.33%
Santos V. A. [88]	72	2	142	UHR-OCT	Custom Neural Network	99.56%
Issarti I. [110]	812	5	812	Pentacam	Logic	99.9%
Shia C. [111]	121	3	121	Pentacam & UHR-OCT	Neural Network	AUC: 0.93, subclinical KCN AUC: 0.99 KCN
Issarti I. [115]	389	2	851	Pentacam	Neural Network	96.56%
Kamiya K. [109]	1824	5	543	Casia 2	CNN	99.1%
Abdelmotaal H. [112]	3218 eyes	3	NA	Pentacam	CNN	95%
Al-Timemy A.H [114]	2136 maps	2	444	Pentacam	Transfer learning	98.3%

* Acc: accuracy, AUC: area under the receiver operator characteristic curve.

## Data Availability

Data are contained within the article.

## References

[1] Guo C., Wang Z., He P., Chen G., Zheng X. (2017). Prevalence, Causes and Social Factors of Visual Impairment among Chinese Adults: Based on a National Survey. Int. J. Environ. Res. Public Health.

[2] Astley R.A., Mursalin M.H., Coburn P.S., Livingston E.T., Nightengale J.W., Bagaruka E., Hunt J.J., Callegan M.C. (2023). Ocular Bacterial Infections: A Ten-Year Survey and Review of Causative Organisms Based on the Oklahoma Experience. Microorganisms.

[3] Jenkins G.R., Yuen H.K., Vogtle L.K. (2015). Experience of Multisensory Environments in Public Space among People with Visual Impairment. Int. J. Environ. Res. Public Health.

[4] Cavazos Quero L., Iranzo Bartolomé J., Cho J. (2021). Accessible Visual Artworks for Blind and Visually Impaired People: Comparing a Multimodal Approach with Tactile Graphics. Electronics.

[5] Khusro S., Shah B., Khan I., Rahman S. (2022). Haptic Feedback to Assist Blind People in Indoor Environment Using Vibration Patterns. Sensors.

[6] Kolačko Š., Predović J., Tomić A., Oršulić V. (2023). Life Quality in Patients with Impaired Visual Acuity Undergoing Intravitreal Medication Applications. Int. J. Environ. Res. Public Health.

[7] Yen C.-Y., Fang I.-M., Hu H.-Y., Weng S.-H. (2022). Association of Visual Impairment with Psychological Distress in Older Adults: A Survey of 105,092 Older People in Taiwan. J. Clin. Med..

[8] Hussain S.F., Heinze N., Gomes R.S.M. (2024). Health and Comorbidities in Minority Ethnic Adults Living with Visual Impairment in the UK. Disabilities.

[9] Choi M., Lee S., Hwang S., Park M., Lee H.-S. (2020). Comparison of Emergency Response Abilities and Evacuation Performance Involving Vulnerable Occupants in Building Fire Situations. Sustainability.

[10] Al-Atawi S., Alghamdi A., Alzahrani K. (2023). The Lifetime Expenditure in People with Keratoconus in Saudi Arabia. Vision.

[11] World Health Organization (2019). World Report on Vision.

[12] Community Medicine for Academics and Lay Learners, WHO Updates Fact Sheet on Blindness and Visual Impairment (11 October 2018). https://communitymedicine4all.com/2018/10/15/who-updates-fact-sheet-on-blindness-and-visual-impairment/.

[13] Walle H., De Runz C., Serres B., Venturini G. (2022). A Survey on Recent Advances in AI and Vision-Based Methods for Helping and Guiding Visually Impaired People. Appl. Sci..

[14] de Freitas M.P., Piai V.A., Farias R.H., Fernandes A.M.R., de Moraes Rossetto A.G., Leithardt V.R.Q. (2022). Artificial Intelligence of Things Applied to Assistive Technology: A Systematic Literature Review. Sensors.

[15] Elmannai W., Elleithy K. (2017). Sensor-Based Assistive Devices for Visually-Impaired People: Current Status, Challenges, and Future Directions. Sensors.

[16] Plikynas D., Žvironas A., Budrionis A., Gudauskis M. (2020). Indoor Navigation Systems for Visually Impaired Persons: Mapping the Features of Existing Technologies to User Needs. Sensors.

[17] Simões W.C.S.S., Machado G.S., Sales A.M.A., de Lucena M.M., Jazdi N., de Lucena V.F. (2020). A Review of Technologies and Techniques for Indoor Navigation Systems for the Visually Impaired. Sensors.

[18] Messaoudi M.D., Menelas B.-A.J., Mcheick H. (2022). Review of Navigation Assistive Tools and Technologies for the Visually Impaired. Sensors.

[19] Wei Z., Qu H., Wang Y., Yuan X., Wu H., Du Y., Han K., Zhang N., Feng Z. (2023). Integrated Sensing and Communication Signals Toward 5G-A and 6G: A Survey. IEEE Internet Things J..

[20] Wang J.-Y., Yang H.-N., Wang J.-B., Lin M., Shi P. (2023). Joint Optimization of Slot Selection and Power Allocation in Integrated Visible Light Communication and Sensing Systems. IEEE Internet Things J..

[21] Zhou B., Wang X., Shen Y., Fan P. (2024). 6-DoF Location-and-Pose Estimation towards Integrated Visible Light Communication and Sensing: Algorithm Design and Performance Limits. IEEE Trans. Signal Process..

[22] Zafar S., Asif M., Bin Ahmad M., Ghazal T.M., Faiz T., Ahmad M., Khan M.A. (2022). Assistive Devices Analysis for Visually Impaired Persons: A Review on Taxonomy. IEEE Access.

[23] Mlynski R., Kozlowski E., Adamczyk J. (2021). Sounds That People with Visual Impairment Want to Experience. Int. J. Environ. Res. Public Health.

[24] El-Taher F.E.-Z., Taha A., Courtney J., Mckeever S. (2021). A Systematic Review of Urban Navigation Systems for Visually Impaired People. Sensors.

[25] Fei Z., Yang E., Hu H., Zhou H. Review of machine vision-based electronic travel aids. Proceedings of the 23rd International Conference on Automation and Computing (ICAC).

[26] Wayband. https://haptic.works/wayband.

[27] Blindsquare. https://blindsquare.com.

[28] Mai C., Xie D., Zeng L., Li Z., Li Z., Qiao Z., Qu Y., Liu G., Li L. (2023). Laser Sensing and Vision Sensing Smart Blind Cane: A Review. Sensors.

[29] Rupam P., Aditi S., Raunak R., Rohit V., Errapa G. (2023). Assistive Device for Deaf, Dumb and Blind using Raspberry-pi. Int. Res. J. Mod. Eng. Technol. Sci..

[30] Ara. https://www.strap.tech/the-device.

[31] WeWalk Smart Cane. https://wewalk.io/en/product/.

[32] NVDA. https://www.nvaccess.org/.

[33] Text to Speech TTS Voice Dream. https://www.voicedream.com/.

[34] Seeing AI. https://www.seeingai.com/.

[35] Aira. https://aira.io/.

[36] GoodMaps. https://goodmaps.com/the-app/.

[37] Be My Eyes. https://www.bemyeyes.com/.

[38] Wayaround. https://www.wayaround.com/.

[39] OrCam MyEye 3 Pro. https://www.orcam.com/en-us/orcam-myeye-3-pro.

[40] Brailliant BI 20X. https://store.humanware.com/hus/brailliant-bi-20x-braille-display.html.

[41] Victor Reader Stream 3. https://store.humanware.com/hus/victor-reader-stream-handheld-media-player.html.

[42] Reveal 16i. https://store.humanware.com/int/reveal-16i-full-hd-digital-magnifier.html.

[43] BrailleNote Touch Plus 32. https://store.humanware.com/int/blindness-braillenote-touch-plus-32.html.

[44] Canute 360 Premium. https://bristolbraille.org/product/canute-360-premium/.

[45] Optelec ClearReader+. https://us.optelec.com/products/cr-ba-g2-us-13m-optelec-clearreader.html.

[46] Envision Glasses. https://www.letsenvision.com/.

[47] DOT Watch 2. https://www.dotincorp.com/61/54?page=8.

[48] DOT Pad. https://www.dotincorp.com/page/32?gbn2=DotPad.

[49] eSight 4. https://www.esighteyewear.com/esight-4/.

[50] BlindShell. https://www.blindshell.com/.

[51] Nearby Explorer. https://tech.aph.org/.

[52] Căilean A.M., Dimian M. (2017). Impact of IEEE 802.15.7 Standard on Visible Light Communications Usage in Automotive Applications. IEEE Commun. Mag..

[53] Matheus L.E.M., Vieira A.B., Vieira L.F.M., Vieira M.A.M., Gnawali O. (2019). Visible Light Communication: Concepts, Applications and Challenges. IEEE Commun. Surv. Tutor..

[54] Rehman S.U., Ullah S., Chong P.H.J., Yongchareon S., Komosny D. (2019). Visible Light Communication: A System Perspective—Overview and Challenges. Sensors.

[55] Cole M., Clayton H., Martin K. (2015). Solid-State Lighting: The New Normal in Lighting. IEEE Trans. Ind. Appl..

[56] Schratz M., Gupta C., Struhs T.J., Gray K. (2016). A New Way to See the Light: Improving Light Quality with Cost-Effective LED Technology. IEEE Ind. Appl. Mag..

[57] James Singh K., Huang Y.-M., Ahmed T., Liu A.-C., Huang Chen S.-W., Liou F.-J., Wu T., Lin C.-C., Chow C.-W., Lin G.-R. (2020). Micro-LED as a Promising Candidate for High-Speed Visible Light Communication. Appl. Sci..

[58] Wang L., Wei Z., Chen C.J., Wang L., Fu H.Y., Zhang L., Chen K.C., Wu M.C., Dong Y., Hao Z. (2021). 1.3 GHz E-O bandwidth GaN-based micro-LED for multi-gigabit visible light communication. Photon. Res..

[59] The European Green Deal-Communication from the Commission to the European Parliament; the European Council, the Council, the European Economic and Social Committee and the Committee of the Regions, Brussels, Belgium, 11 December 2019. https://commission.europa.eu/publications/communication-european-green-deal_en.

[60] Hussein A.T., Alresheedi M.T., Elmirghani J.M.H. (2015). 20 Gb/s Mobile Indoor Visible Light Communication System Employing Beam Steering and Computer Generated Holograms. J. Light. Technol..

[61] Bian R., Tavakkolnia I., Haas H. (2019). 15.73 Gb/s Visible Light Communication with Off-the-Shelf LEDs. J. Light. Technol..

[62] Tsonev D., Videv S., Haas H. (2015). Towards a 100 Gb/s visible light wireless access network. Opt. Express.

[63] Dastgheib M.A., Beyranvand H., Salehi J.A., Maier M. (2018). Mobility-Aware Resource Allocation in VLC Networks Using T-Step Look-Ahead Policy. J. Light. Technol..

[64] Al-Ahmadi S., Maraqa O., Uysal M., Sait S.M. (2018). Multi-User Visible Light Communications: State-of-the-Art and Future Directions. IEEE Access.

[65] Mohammedi Merah M., Guan H., Chassagne L. (2019). Experimental Multi-User Visible Light Communication Attocell Using Multiband Carrierless Amplitude and Phase Modulation. IEEE Access.

[66] Abuella H., Elamassie M., Uysal M., Xu Z., Serpedin E., Qaraqe K.A., Ekin S. (2021). Hybrid RF/VLC Systems: A Comprehensive Survey on Network Topologies, Performance Analyses, Applications, and Future Directions. IEEE Access.

[67] Noor-A-Rahim M., Liu Z., Lee H., Khyam M.O., He J., Pesch D., Poor H.V. (2022). 6G for Vehicle-to-Everything (V2X) Communications: Enabling Technologies, Challenges, and Opportunities. Proc. IEEE.

[68] de Souza Lopes C.H., Andrade T.P.V., Pereira L.A.M., Bogoni A., Conforti E., Sodré A.C. (2023). Implementation of a Hybrid FiWi System Using FSO, VLC and mm-Waves Toward 6G Applications. IEEE Photonics Technol. Lett..

[69] LiFi Technology: Unleashing the Power of Light for Enhanced Connectivity. https://www.oledcomm.net/.

[70] Avătămăniţei S.-A., Căilean A.-M., Done A., Căpitan A., Popa V. Indoor Visible Light Communications demonstration: University Campus Radio Station transmitted through the lighting system. Proceedings of the 2019 6th International Symposium on Electrical and Electronics Engineering (ISEEE).

[71] Căilean A.M., Dimian M. (2017). Current Challenges for Visible Light Communications Usage in Vehicle Applications: A Survey. IEEE Commun. Surv. Tutor..

[72] Căilean A.-M., Beguni C., Avătămăniței S.-A., Dimian M., Popa V. (2022). Design, Implementation and Experimental Investigation of a Pedestrian Street Crossing Assistance System Based on Visible Light Communications. Sensors.

[73] Căilean A.-M., Avătămăniței S.-A., Beguni C. (2024). Driving toward Connectivity: Vehicular Visible Light Communications Receiver with Adaptive Field of View for Enhanced Noise Resilience and Mobility. Sensors.

[74] Do T.-H., Yoo M. (2016). An in-Depth Survey of Visible Light Communication Based Positioning Systems. Sensors.

[75] Zhuang Y., Hua L., Qi L., Yang J., Cao P., Cao Y., Haas H. (2018). A Survey of Positioning Systems Using Visible LED Lights. IEEE Commun. Surv. Tutor..

[76] Liang Q., Sun Y., Liu C., Liu M., Wang L. (2022). LedMapper: Toward Efficient and Accurate LED Mapping for Visible Light Positioning at Scale. IEEE Trans. Instrum. Meas..

[77] Cahyadi W.A., Kim Y.-H., Chung Y.-H., Ghassemlooy Z. Efficient road surface detection using visible light communication. Proceedings of the 2015 Seventh International Conference on Ubiquitous and Future Networks.

[78] Abuella H., Miramirkhani F., Ekin S., Uysal M., Ahmed S. (2019). ViLDAR—Visible Light Sensing-Based Speed Estimation Using Vehicle Headlamps. IEEE Trans. Veh. Technol..

[79] Alsalami F.M., Ahmad Z., Zvanovec S., Haigh P.A., Haas O.C.L., Rajbhandari S. (2019). Indoor Intruder Tracking Using Visible Light Communications. Sensors.

[80] Zhou H., Zhang M., Ren X. (2023). Design and Implementation of Wireless Optical Access System for VLC-IoT Networks. J. Light. Technol..

[81] Wang F., Yang F., Pan C., Song J., Han Z. (2022). Joint Illumination and Communication Optimization in Indoor VLC for IoT Applications. IEEE Internet Things J..

[82] Shi J., Niu W., Ha Y., Xu Z., Li Z., Yu S., Chi N. (2022). AI-Enabled Intelligent Visible Light Communications: Challenges, Progress, and Future. Photonics.

[83] Guo L.L., Tian L., Cao K., Li Y.X., Li N., Yang W.Q., Jie Y. (2021). Comparison of the Morphological and Biomechanical Characteristics of Keratoconus, Forme Fruste Keratoconus, and Normal Corneas. Semin. Ophthalmol..

[84] Jhanji V., Sharma N., Vajpayee R.B. (2011). Management of Keratoconus: Current Scenario. Br. J. Ophthalmol..

[85] Marschall S., Gawish A., Feng Y., Sorbara L., Fieguth P., Bizheva K. (2013). In-Vivo Imaging of Keratoconic Corneas Using High-Speed High-Resolution Swept-Source OCT. Optics InfoBase Conference Papers.

[86] Gomes J.A.P., Tan D., Rapuano C.J., Belin M.W., Ambrósio R., Guell J.L., Malecaze F., Nishida K., Sangwan V.S. (2015). Global Consensus on Keratoconus and Ectatic Diseases. Cornea.

[87] Ang M., Baskaran M., Werkmeister R.M., Chua J., Schmidl D., Aranha dos Santos V., Garhöfer G., Mehta J.S., Schmetterer L. (2018). Anterior Segment Optical Coherence Tomography. Prog. Retin. Eye Res..

[88] dos Santos V.A., Schmetterer L., Stegmann H., Pfister M., Messner A., Schmidinger G., Garhofer G., Werkmeister R.M. (2019). CorneaNet: Fast Segmentation of Cornea OCT Scans of Healthy and Keratoconic Eyes Using Deep Learning. Biomed. Opt. Express.

[89] Williams D., Zheng Y., Davey P.G., Bao F., Shen M., Elsheikh A. (2016). Reconstruction of 3D Surface Maps from Anterior Segment Optical Coherence Tomography Images Using Graph Theory and Genetic Algorithms. Biomed. Signal Process. Control.

[90] LaRocca F., Chiu S.J., McNabb R.P., Kuo A.N., Izatt J.A., Farsiu S. (2011). Robust Automatic Segmentation of Corneal Layer Boundaries in SDOCT Images Using Graph Theory and Dynamic Programming. Biomed. Opt. Express.

[91] Iskander D.R., Morelande M.R., Collins M.J., Davis B. (2002). Modeling of Corneal Surfaces with Radial Polynomials. IEEE Trans. Biomed. Eng..

[92] Schmoll T., Unterhuber A., Kolbitsch C., Le T., Stingl A., Leitgeb R. (2012). Precise Thickness Measurements of Bowman’s Layer, Epithelium, and Tear Film. Optom. Vis. Sci..

[93] Jahromi M.K., Kafieh R., Rabbani H., Dehnavi A.M., Peyman A., Hajizadeh F., Ommani M. (2014). An Automatic Algorithm for Segmentation of the Boundaries of Corneal Layers in Optical Coherence Tomography Images Using Gaussian Mixture Model. J. Med. Signals Sens..

[94] Zhang T., Elazab A., Wang X., Jia F., Wu J., Li G., Hu Q. (2017). A Novel Technique for Robust and Fast Segmentation of Corneal Layer Interfaces Based on Spectral-Domain Optical Coherence Tomography Imaging. IEEE Access.

[95] Gayathri S., Gopi V.P., Palanisamy P. (2020). A Lightweight CNN for Diabetic Retinopathy Classification from Fundus Images. Biomed. Signal Process. Control.

[96] Li L., Xu M., Liu H., Li Y., Wang X., Jiang L., Wang Z., Fan X., Wang N. (2020). A Large-Scale Database and a CNN Model for Attention-Based Glaucoma Detection. IEEE Trans. Med. Imaging.

[97] Thomas A., Harikrishnan P.M., Krishna A.K., Palanisamy P., Gopi V.P. (2021). Automated Detection of Age-Related Macular Degeneration from OCT Images Using Multipath CNN. J. Comput. Sci. Eng..

[98] Wang L., Shen M., Chang Q., Shi C., Chen Y., Zhou Y., Zhang Y., Pu J., Chen H. (2021). Automated Delineation of Corneal Layers on OCT Images Using a Boundary-Guided CNN. Pattern Recognit..

[99] Sonar H., Kadam A., Bhoir P., Joshi B. (2020). Detection of Keratoconus Disease. ITM Web Conf..

[100] Chen X., Zhao J., Iselin K.C., Borroni D., Romano D., Gokul A., McGhee C.N.J., Zhao Y., Sedaghat M.R., Momeni-Moghaddam H. (2021). Keratoconus Detection of Changes Using Deep Learning of Colour-Coded Maps. BMJ Open Ophthalmol..

[101] Fırat M., Çankaya C., Çınar A., Tuncer T. (2022). Automatic Detection of Keratoconus on Pentacam Images Using Feature Selection Based on Deep Learning. Int. J. Imaging Syst. Technol..

[102] Lavric A., Valentin P. (2019). KeratoDetect: Keratoconus Detection Algorithm Using Convolutional Neural Networks. Comput. Intell. Neurosci..

[103] User Manual CASIA. https://simovision.com/assets/Uploads/User-Manual-Tomey-CASIA2-EN.pdf.

[104] Bamdad S., Sedaghat M.R., Yasemi M., Vahedi A. (2020). Sensitivity and Specificity of Belin Ambrosio Enhanced Ectasia Display in Early Diagnosis of Keratoconus. J. Ophthalmol..

[105] Vaishya R., Javaid M., Khan I.H., Haleem A. (2020). Artificial Intelligence (AI) Applications for COVID-19 Pandemic. Diabetes Metab. Syndr. Clin. Res. Rev..

[106] Choi R.Y., Coyner A.S., Kalpathy-Cramer J., Chiang M.F., Peter Campbell J. (2020). Introduction to Machine Learning, Neural Networks, and Deep Learning. Transl. Vis. Sci. Technol..

[107] Rozema J.J., Rodriguez P., Ruiz Hidalgo I., Navarro R., Tassignon M.J., Koppen C. (2017). SyntEyes KTC: Higher Order Statistical Eye Model for Developing Keratoconus. Ophthalmic Physiol. Opt..

[108] Kuo B.I., Chang W.Y., Liao T.S., Liu F.Y., Liu H.Y., Chu H.S., Chen W.L., Hu F.R., Yen J.Y., Wang I.J. (2020). Keratoconus Screening Based on Deep Learning Approach of Corneal Topography. Transl. Vis. Sci. Technol..

[109] Kamiya K., Ayatsuka Y., Kato Y., Fujimura F., Takahashi M., Shoji N., Mori Y., Miyata K. (2019). Keratoconus Detection Using Deep Learning of Colour-Coded Maps with Anterior Segment Optical Coherence Tomography: A Diagnostic Accuracy Study. BMJ Open.

[110] Issarti I., Consejo A., Jiménez-García M., Kreps E.O., Koppen C., Rozema J.J. (2020). Logistic Index for Keratoconus Detection and Severity Scoring (Logik). Comput. Biol. Med..

[111] Shi C., Wang M., Zhu T., Zhang Y., Ye Y., Jiang J., Chen S., Lu F., Shen M. (2020). Machine Learning Helps Improve Diagnostic Ability of Subclinical Keratoconus Using Scheimpflug and OCT Imaging Modalities. Eye Vis..

[112] Abdelmotaal H., Mostafa M.M., Mostafa A.N.R., Mohamed A., Abdelazeem K. (2020). Classification of Color-Coded Scheimpflug Camera Corneal Tomography Images Using Deep Learning. Transl. Vis. Sci. Technol..

[113] Zéboulon P., Debellemanière G., Bouvet M., Gatinel D. (2020). Corneal Topography Raw Data Classification Using a Convolutional Neural Network. Am. J. Ophthalmol..

[114] Al-Timemy A.H., Ghaeb N.H., Mosa Z.M., Escudero J. (2021). Deep Transfer Learning for Improved Detection of Keratoconus Using Corneal Topographic Maps. Cogn. Comput..

[115] Issarti I., Consejo A., Jiménez-García M., Hershko S., Koppen C., Rozema J.J. (2019). Computer Aided Diagnosis for Suspect Keratoconus Detection. Comput. Biol. Med..

[116] Liu X., Makino H., Kobayashi S., Maeda Y. An Indoor Guidance System for the Blind using Fluorescent Lights-Relationship between Receiving Signal and Walking Speed. Proceedings of the 2006 International Conference of the IEEE Engineering in Medicine and Biology Society.

[117] Liu X., Makino H., Kobayashi S., Maeda Y. Design of an Indoor Self-Positioning System for the Visually Impaired-Simulation with RFID and Bluetooth in a Visible Light Communication System. Proceedings of the 2007 29th Annual International Conference of the IEEE Engineering in Medicine and Biology Society.

[118] Liu X., Makino H., Maeda Y. Basic study on indoor location estimation using Visible Light Communication platform. Proceedings of the 2008 30th Annual International Conference of the IEEE Engineering in Medicine and Biology Society.

[119] Suzuki K., Fujita M., Hayashi Y., Fukuzono K. (2008). A study on visually impaired person’s support system utilizing visible light communication technology at signalized intersections. Int. J. ITS Res..

[120] Araki T., Tatsumi H., Suzuki T., Yamada K. Application of LED visible light communication signaling for the visually impaired. Proceedings of the 2010 IEEE International Conference on Systems, Man and Cybernetics.

[121] Funahashi A., Kobayashi K., Okada H., Katayama M. i-LightHouse: A Visible Light Communication system for the visually impaired. Proceedings of the 2011 IEEE 22nd International Symposium on Personal, Indoor and Mobile Radio Communications.

[122] Nakajima M., Haruyama S. Indoor navigation system for visually impaired people using visible light communication and compensated geomagnetic sensing. Proceedings of the 2012 1st IEEE International Conference on Communications in China (ICCC).

[123] Araki T., Suzuki T. Fuzzy timing passwords for providing easy user authentication to disable persons and their application to visible light communication. Proceedings of the World Automation Congress 2012.

[124] Kii H., Murata Y., Oshiba S., Nagai Y., Watanabe H., Iki S., Morimoto K. Accessible Optical Wireless Pedestrian-Support Systems for Individuals with Visual Impairment. Proceedings of the 2014 IIAI 3rd International Conference on Advanced Applied Informatics.

[125] Oshiba S., Iki S., Yabuchi J., Mizutani Y., Kawabata K., Nakagawa K., Morimoto K. Visibility evaluation experiments of optical wireless pedestrian-support system using self-illuminating bollard. Proceedings of the 2016 IEEE/ACIS 15th International Conference on Computer and Information Science (ICIS).

[126] Okuda K., Oda S., Nakamura T., Uemura W. Information delivery tactile pavings using visible light communication. Proceedings of the 2014 IEEE International Conference on Consumer Electronics (ICCE).

[127] Jayakody A., Meegama C.I., Pinnawalage H.U., Muwenwella R.M.H.N., Dalpathado S.C. AVII [Assist Vision Impaired Individual]: An Intelligent Indoor Navigation System for the Vision Impaired Individuals with VLC. Proceedings of the 2016 IEEE International Conference on Information and Automation for Sustainability (ICIAfS).

[128] Perez-Jimenez R., Rabadan J., Guerra V., Aguiar L., Rufo J. Fundamentals of Indoor Vlp: Providing Autonomous Mobility for Visually Impaired People. Proceedings of the 2017 International Conference and Workshop on Bioinspired Intelligence (IWOBI).

[129] Audomphon A., Apavatjrut A. Smart Glasses for Sign Reading as Mobility Aids for the Blind Using a Light Communication System. Proceedings of the 2020 17th International Conference on Electrical Engineering/Electronics, Computer, Telecommunications and Information Technology (ECTI-CON).

[130] Darlis A.R., Jambola L., Hadyansyah T. (2020). Light follower systems for visually impaired using visible light communication. TELKOMNIKA (Telecommun. Comput. Electron. Control).

[131] Rithuan M.N., Zain A.S., Nawawi N.M. (2020). Indoor Navigation System for Vision Impairment People through Visible Light Communications. IOP Conf. Ser. Mater. Sci. Eng..

[132] Srinithi P., Kalpanadevi S., Rekha P., Divya N., Rajkumar M., Aathiba D. A Novel Paradigm of Indoor Navigation System using Li-Fi Technology. Proceedings of the 2023 2nd International Conference on Automation, Computing and Renewable Systems (ICACRS).

[133] Adoptante E.B., Cadag K.D., Lualhati V.R., Torregoza M.L.D.R., Abad A.C. Audio multicast by Visible Light Communication for location information for the visually impaired. Proceedings of the 2015 International Conference on Humanoid, Nanotechnology, Information Technology, Communication and Control, Environment and Management (HNICEM).

[134] Pradnya M., Tadwalkar P.M. (2017). Visible Light Communication for Visually Impaired People using Sustainable LEDs. Int. J. Trend Sci. Res. Dev..

[135] Pravin M., Sundararajan T.V.P. VLC Based Indoor Blind Navigation System. Proceedings of the 2018 9th International Conference on Computing, Communication and Networking Technologies (ICCCNT).

[136] Botre M.R., Askhedkar A.R. LiFi and Voice Based Indoor Navigation System for Visually Impaired People. Proceedings of the 2019 IEEE Pune Section International Conference (PuneCon).

[137] Nikhil K., Kalyan I.S.P., Sagar J., Rohit M.S., Nesasudha M. Li-Fi Based Smart Indoor Navigation System for Visually Impaired People. Proceedings of the 2019 2nd International Conference on Signal Processing and Communication (ICSPC).

[138] Manimaran D., Anu M., Christina S.F. Integration of Indoor and Outdoor Voice Based Navigation Detection using Light based Communication (Lifi) & IoT. Proceedings of the 2021 5th International Conference on Computing Methodologies and Communication (ICCMC).

[139] Căilean A.-M., Avătămăniței S.-A., Beguni C., Zadobrischi E., Dimian M., Popa V. (2023). Visible Light Communications-Based Assistance System for the Blind and Visually Impaired: Design, Implementation, and Intensive Experimental Evaluation in a Real-Life Situation. Sensors.

[140] Căilean A.-M., Avătămăniței S.-A., Beguni C. Design and Experimental Evaluation of a Visible Light Communications-Based Smart Backpack for Visually Impaired Persons’ Assistance. Proceedings of the 2023 31st Telecommunications Forum (TELFOR).

[141] Căilean A.-M., Beguni C., Avătămăniței S.-A., Dimian M., Chassagne L., Béchadergue B. Experimental Evaluation of an Indoor Visible Light Communications System in Light Dimming Conditions. Proceedings of the 2023 31st Telecommunications Forum (TELFOR).

[142] Căilean A.-M., Avătămăniței S.-A., Beguni C., Zadobrischi E., Dimian M. Lighting Efficiency: Using Visible Light Communications Technology for Enhanced Energy Management in Built Environment and Beyond. Proceedings of the 2024 International Conference on Development and Application Systems (DAS).

